# Supplementary material for: Situation analysis for delivering integrated comprehensive sexual and reproductive health services in humanitarian crisis condition for Rohingya refugees in Cox’s Bazar, Bangladesh: protocol for a mixed-method study
Source: BMJ Open. 2019 Jul 3;9(7):e028340. doi: 10.1136/bmjopen-2018-028340 (PMC6615844; doi:10.1136/bmjopen-2018-028340)
Supplement: Supplementary file 1 [file bmjopen-2018-028340supp001.pdf]

Situation Analysis for Delivering Integrated Comprehensive SRHR Services for Rohingya Refugees in Cox's Bazar, Bangladesh

Facility Assessment

Conducted by
BRAC James P Grant School of Public Health (JPGSPH), BRAC University
Funded by
World Health Organization (WHO)

Questionnaire and Checklists
(For Health Facilities)

A1. Unique identification number of the facility	
A2. Serial number of the facility	
A3. Name of the Upazila	
A4. Name of the camp/area	
A5. Name of the health facility	
A6. Date of the assessment	
A7. Starting time of the assessment	
A8. Ending time of the assessment	
A9. Name of the Respondent, Designation & Cell number	
A10. Name of the assessor	
A11. Signature of the assessor	

1. General Information

1.1 Types of the facility (Please circle the appropriate one)

a. Community clinic
b. Health post
c. Primary Health Center (PHC)
d. National non-government organization (NNGO) Hospital
e. International non-government organization (INGO)Hospital
f. Other (Specify)

1.2 Management of the facility (Please circle the appropriate one)

a. Government organization (GO) (Specify) ____________________________
b. National non-government organization (NNGO) (Specify) _________________________
c. International non-government organization (INGO) (Specify) ______________________
d. Ownership by more than one organization (Specify) (Specify) ______________________
e. Other (Specify) _______________________

1.3 Source of external Financial/resource support if applicable (Please circle the appropriate one)

a. Government organization (GO) (Specify) ____________________________
b. National non-government organization (NNGO) (Specify) _________________________
c. International non-government organization (INGO) (Specify) ________________________
d. Multi-donor (Specify) _________________________
e. Other (Specify) _________________________

1.4 How many days per week is the facility opened? (Please circle the appropriate one)

a. 7 days a week
b. 6 days a week
c. 5 days a week
d. Others (Specify)

1.5 What is your Working hours? (Please circle the appropriate one)

a. 24 hours
b. 9 am - 4 pm
   Other (specify

1.6 Estimated number of population served by your facility: _________

2. Health service information

2.1 Availability of sexual and reproductive health services

Serial no.	Name of the services	Code<br><br>1 = Yes<br>0 = Not available	Designation of the service provider/s<br>Code: 1, 2, 3, 4, 5, 6, 7, 8, 9, 97<br>*(for the details of this code follow the attached codebook)*	If the service is not available, then do you refer the patients?<br>Code<br>1 = Yes<br>0 = No	If yes, write the referred place/s<br>*(Most frequently referred)*
1.	ANC				
1.1	**Obstetric and foetal assessment** –<br>• Maternal weight<br>• BP measurement<br>• Oedema<br>• Fundal height<br>• Foetal heartbeat				
1.2	**Screening and tests**<br>• Urinalysis<br>• Hb estimation<br>• Blood grouping and Rh typing<br>• Testing for HIV, syphilis<br>• Blood sugar<br>• Ultrasonogram (referred cases for suspicion of low foetal growth				
1.3	**Identify & manage obstetric emergencies**<br>• Pre/eclampsia<br>• Ante-partum Haemorrhage<br>• Abdominal pain<br>• Premature rupture of membranes				
1.4	**Health education, advice, and counselling** (Nutrition,				

Serial no.	Name of the services	Code<br><br>1 = Yes<br>0 = Not available	Designation of the service provider/s<br>Code: 1, 2, 3, 4, 5, 6, 7, 8, 9, 97<br>*(for the details of this code follow the attached codebook)*	If the service is not available, then do you refer the patients?<br>Code<br>1 = Yes<br>0 = No	If yes, write the referred place/s<br>*(Most frequently referred)*
	Complications of pregnancy / danger sign, Rest, Birth plan and emergency plan)				
2.	Eclampsia Management				
3.	PPH (Postpartum Hemorrhage) Management				
4.	Maternal Immunization				
5.	Delivery Care<br>• Normal Vaginal Delivery<br>• C-section<br>• Deliveries that require vacuum extraction assistance				
6.	Initial stabilization of obstetric emergency before referral				
7.	Blood transfusion				
8.	Essential new born care				
9.	Post Natal Care				
9.1	Counselling on postnatal care, breastfeeding, etc.				

Serial no.	Name of the services	Code<br><br>1 = Yes<br>0 = Not available	Designation of the service provider/s<br>Code: 1, 2, 3, 4, 5, 6, 7, 8, 9, 97<br>*(for the details of this code follow the attached codebook)*	If the service is not available, then do you refer the patients?<br>Code<br>1 = Yes<br>0 = No	If yes, write the referred place/s<br>*(Most frequently referred)*
9.2	Post-Natal clinical history (pain, fever, haemorrhage)				
9.3	Identification and management of post-natal complications:<br>-Anaemia<br>-Puerperal psychosis				
9.4	Identification and management of obstetric complications:<br>-Haemorrhage<br>-Puerperal infection/sepsis				
9.5	Supply of Iron and Folic Acid				
9.6	Counselling and Provision of FP methods				
10.	Family Planning				
11.	FP services: Pill				
12.	FP services: Male Condoms				
13.	Emergency Contraception				
14.	FP services: Injection				
15.	FP services: Implant/Norplant				
16.	FP services: IUD				

Serial no.	Name of the services	Code<br><br>1 = Yes<br>0 = Not available	Designation of the service provider/s<br>Code: 1, 2, 3, 4, 5, 6, 7, 8, 9, 97<br>*(for the details of this code follow the attached codebook)*	If the service is not available, then do you refer the patients?<br>Code<br>1 = Yes<br>0 = No	If yes, write the referred place/s<br>*(Most frequently referred)*
17.	FP services: Vasectomy				
18.	FP services: Tubectomy				
19.	STD/RTI Management				
20.	HIV AIDS Counseling				
21.	HIV AIDS Testing				
22.	Prevention of maternal –to-child transmission of HIV (PMTCT) *(one kind of medicine to stop transmitting HIV to the newborn)*				
23.	Menstrual regulation (MR)				
24.	Management of miscarriage and complications of abortions				
25.	Clinical Management of Rape (CMR)				
26.	Management of Psychosocial issues				
27.	Adolescent friendly health services (counseling on Sex education/ sexual health care)				
28.	Adolescent friendly health services				

Serial no.	Name of the services	Code<br><br>1 = Yes<br>0 = Not available	Designation of the service provider/s<br>Code: 1, 2, 3, 4, 5, 6, 7, 8, 9, 97<br>*(for the details of this code follow the attached codebook)*	If the service is not available, then do you refer the patients?<br>Code<br>1 = Yes<br>0 = No	If yes, write the referred place/s<br>*(Most frequently referred)*
	(counseling on FP services)				
29.	Adolescent friendly health services (counseling on HIV services)				
30.	Others				

2.2 If you don't provide the SRHR services, please mention the reason for not providing the SRHR services.

____
____
____

2.3 Human Resources: Key Staff information

Serial no.	Categories	No of staff	Job Nature<br>Code<br>1= Fulltime<br>2= Part-time<br>3= On-call
1.	Gynecologists / Gynae Consultant		
2.	Anesthesiologist		
3.	Residential Medical Officer (RMO)		

Serial no.	Categories	No of staff	Job Nature Code<br>1= Fulltime<br>2= Part-time<br>3= On-call
4.	Medical Officer		
5.	Medical Assistant		
6.	SACMO		
7.	Health Inspector		
8.	Nurse		
9.	Midwife		
10.	Trained Birth Attendants (TBAs)		
11.	Family Welfare Assistant (FWA)		
12.	Community Health Care Provider (CHCP)		
13.	Psycho-social Counsellor		
14.	Laboratory technicians/ Medical Technologist		
97.	Others (Specify)		

2.4 Training

If any of your staff received any of the following training:

Serial No.	Types of Training (RECEIVED IN THE PAST Three MONTHS)	Code<br>1 = Yes<br>0 = No	Who received it (Doctor, Nurse, Midwifes etc?) Code: 1 – 20, 97<br>*(for the details of this code follow the attached codebook)*
1.	Helping Babies Breathe		
2.	Emergency Response		
3.	Helping Mothers Survive		

4.	Gender Based Violence		
5.	Clinical Management of Rape (CMR)		
6.	OGSB Training - FP (Long Acting & Permanent)		
7.	EmONC		
8.	OGSB Training - Infection Prevention Control		
9.	OGSB Training – RTI & STI		
10.	OGSB Training – Management & Care of Maternal & Child Nutrition		
11.	ANC Package		
12.	MR & PAC		
13.	Orientation on community distribution of misoprostol		
14.	MISP Training for Coordinator		
15.	OGSB Training – Safe Delivery Practice		
16.	Labor Room Protocol		
17.	Cervical Cancer Screening		
18.	FP Counselling		
19.	IUD		
20.	Implant		
21.	PPIUD		
22.	Others		

2.4.1 Do you think is there any other training that you/your staff need to receive?

a. No = 0
b. Yes = 1 If Yes, specify ______________________________________

2.5 Information on total number of services

2.5.1 On average total number of Outpatient served in a day?

2.5.2 Total number of inpatient served in a month? (Last month/3 month average)

2.5.3 Information on total number of services

Serial no.	Services and Quantity	July 2018	August 2018	September 2018
1.	No. of total patients served			
2.	No. of ANC			
3.	Eclampsia Management			
4.	PPH (Postpartum Hemorrhage) Management			
5.	Total delivery			
	Normal Delivery			
	C section			
6.	No. of PNC			
7.	Blood Transfusion			
8.	No. of FP counseling			
9.	FP services: Pill			
10.	FP services: Condom			
11.	FP services: Injection			
12.	FP services: Implant/Norplant			
13.	FP services: IUD			
14.	FP services: Vasectomy			
15.	FP services: Tubectomy			
16.	No. of STD/RTI screening			
17.	No. of STD/RTI treatment			
18.	No of HIV/AIDS counselling			
19.	No of HIV/AIDS screening			

Serial no.	Services and Quantity	July 2018	August 2018	September 2018
20.	No. of MR service			
21.	No. of management of miscarriage and complications of abortions			
22.	No of Post rape treatment			
23.	No. of adolescent health service			
24.	Total no. of referred patients			
25.	Other (Specify			

2.6 Availability of ambulance service

2.6.1 Is there any ambulance services in your facility?

a. Yes = 1
b. No = 0

2.6.2 If yes, then how many do you have? [ ]

2.6.3 If No, Then do you have access to other's (private/NGO/UN/govt.) ambulance services that you could use?

a. Yes = 1, Specify whose____________________________
b. No = 0

2.6.4 When do you refer patients, how do you transport them to the referred place? (Mode of transportation)

____ (Free Listing)

2.7 Structure of the facility /Availability of rooms (related to SRH)

Serial no.	Name of department/room	Code<br>1 = Yes<br>0 = Not available	Remark
1.	Emergency room		
2.	Consultant's room		
3.	Counselling room		
4.	Patient's waiting place		
5.	ANC/PNC room		
6.	Labor Room		
7.	Labor Ward		
8.	Women Ward		
9.	VIA/PAC room		
10.	Room for USG		
11.	Laboratory for lab tests		
12.	Room for blood transfusion		
13.	Maternity OPD		
14.	Breast feeding corner in OPD		
15.	General Operation Theatre		
16.	Dispensary		
17.	Other (Specify)		

2.8 Number of maternity beds at the facility

2.9 Number of beds occupied by the patients on average

3. Equipment, drugs and supplies

Serial No	KIT Number & Name	Received? Yes = 1 No = 0	If yes, How many? (Boxes)	If No, Then Why?	If not a UNFPA KIT receiver then how do you procure/ source those drugs and supplies
1	Kit 1: Condoms				
2	Kit 2: Clean Delivery, Individual				
3	Kit 3: Post Rape Treatment				
4	Kit 4 Oral And Injectable Contraception				
5	Kit 5: Treatment Of Sexually Transmitted Infections				
6	Kit 6: Clinical Delivery Assistance				
7	Kit 7: Intrauterine Device				
8	Kit 8: Management Of Miscarriage And Complications Of Abortion				
9	Kit 9: Suture Of Tears (Cervical & Vaginal Examination)				
10	Kit 10: Vacuum Extraction Delivery				
11	Kit 11: Referral Level Kit For Reproductive Health				
12	Kit 12 Blood Transfusion				

Infrastructure

a. Standard precautions for infection prevention (ref: SARA reference manual of WHO)

Serial no.	Activities	Code<br>1 = Yes<br>0 = Not available	Remarks *(How?)*
1.	Appropriate storage of sharp object wastes (sharps box/container)		
2.	Appropriate storage of infectious waste (waste receptacle with lid and plastic bin liner)		
3.	Safe final disposal of sharp objects		
4.	Safe final disposal of infectious wastes		

ANNEXURE

Code Book:

2.1 Health Service Information on SRHR Designation of the service provider/s Code: 1 – 20, 97 *(for the details please follow below mentioned list)*

1 = Gynecologists / Gynae Consultant

2 = Anesthesiologist

3 = Residential Medical Officer (RMO)

4 = Medical Officer

5 = Medical Assistant

6 = SACMO

7 = Health Inspector

8 = Nurse

9 = Midwife

10 = Trained Birth Attendants (TBA)

11 = other community health workers (FWA)

12 = CHCP

13 = Counsellor

14 = Laboratory technicians/ Medical Technologist

97 = Other (specify)
