# Supplementary material for: Situation analysis for delivering integrated comprehensive sexual and reproductive health services in humanitarian crisis condition for Rohingya refugees in Cox’s Bazar, Bangladesh: protocol for a mixed-method study
Source: BMJ Open. 2019 Jul 3;9(7):e028340. doi: 10.1136/bmjopen-2018-028340 (PMC6615844; doi:10.1136/bmjopen-2018-028340)
Supplement: Author's manuscript [file bmjopen-2018-028340.draft_revisions.pdf]

BMJ Open is committed to open peer review. As part of this commitment we make the peer review history of every article we publish publicly available.

When an article is published we post the peer reviewers' comments and the authors' responses online. We also post the versions of the paper that were used during peer review. These are the versions that the peer review comments apply to.

The versions of the paper that follow are the versions that were submitted during the peer review process. They are not the versions of record or the final published versions. They should not be cited or distributed as the published version of this manuscript.

BMJ Open is an open access journal and the full, final, typeset and author-corrected version of record of the manuscript is available on our site with no access controls, subscription charges or pay-per-view fees (http://bmjopen.bmj.com).

If you have any questions on BMJ Open's open peer review process please email info.bmjopen@bmj.com

BMJ Open

Situation analysis for delivering integrated comprehensive sexual and reproductive health services in humanitarian crisis condition for Rohingya refugees in Cox's Bazar, Bangladesh: protocol for a mixed-method study

Journal:	*BMJ Open*
Manuscript ID	bmjopen-2018-028340
Article Type:	Protocol
Date Submitted by the Author:	03-Dec-2018

Complete List of Authors:	AHMED, RUSHDIA; BRAC University James P Grant School of Public Health<br>Farnaz, Nadia; BRAC University James P Grant School of Public Health<br>Aktar, Bachera; BRAC University James P Grant School of Public Health<br>Hassan, Raafat; BRAC University James P Grant School of Public Health<br>Shafique , Sharid ; BRAC University James P Grant School of Public Health<br>Ray, Pushpita ; BRAC University James P Grant School of Public Health<br>Awal, Abdul ; BRAC University James P Grant School of Public Health<br>Rahman, Atiya; BRAC University James P Grant School of Public Health<br>Urbaniak , Veronique; World Health Organization , Reproductive Health and Research<br>Kobeissi, Loulou; World Health Organization , Reproductive Health and Research<br>Rosie, Jeffries; World Health Organization , Health Sector Coordination Office<br>Say, Lale; World Health Organization, Reproductive Health and Research<br>Hasan, Md Tanvir; BRAC University James P Grant School of Public Health<br>Quayyum , Zahidul ; BRAC University James P Grant School of Public Health<br>Rashid, Sabina Faiz; BRAC University James P Grant School of Public Health

Keywords:	Sexual and reproductive health, humanitarian crises, Situation analysis, Rohingya refugees, Women, Adolescent girls

SCHOLARONE™
Manuscripts

Situation analysis for delivering integrated comprehensive sexual and reproductive health services in humanitarian crisis condition for Rohingya refugees in Cox's Bazar, Bangladesh: protocol for a mixed-method study

Rushdia Ahmed[1], Nadia Farnaz[1], Bachera Aktar[1], Raafat Hassan[1], Sharid Bin Shafique[1], Pushpita Ray[1], Abdul Awal[1], Atiya Rahman[1], Veronique Urbaniak[2], Loulou Hassan Kobeissi[2], Jeffries Rosie[3], Lale Say[2], Md Tanvir Hasan[1], Zahidul Quayyum[1], Sabina Faiz Rashid[1]

[1] BRAC James P Grant School of Public Health, BRAC University, Dhaka, Bangladesh
[2] Department of Reproductive Health Research, World Health Organization, Geneva, Switzerland
[3] Health Sector Coordination Office, World Health Organization, Cox's Bazar, Bangladesh

*Corresponding author: Rushdia Ahmed (RA)

Contact Address: 68, Shaheed Tajuddin Ahmed Sarani, Mohakhali, Dhaka, Bangladesh – 1212. Telephone number: +880 178 174 2055.

Email: ahmed.rushdia@yahoo.com

For peer review only

For peer review only - http://bmjopen.bmj.com/site/about/guidelines.xhtml

ABSTRACT

Introduction

Rohingya diaspora are one of the most vulnerable groups seeking refuge in camps of Cox's Bazar, Bangladesh, arising an acute humanitarian crisis. More than half of the Rohingya refugees are women and adolescent girls requiring quality sexual and reproductive health services. Although minimum initial service package of sexual and reproductive health (SRH) are being rendered, World Health Organization is aiming to provide integrated comprehensive SRH services to meet the immediate needs of this most vulnerable group. For sustainable and successful implementation of such comprehensive SRH service package, a critical first step is to undertake a situation analysis and understand the current dimensions, and capture the lessons learned. This situation analysis is pertinent in current humanitarian condition and will provide an overview of the needs, availability and delivery of SRH services for adolescent girls and women, facility readiness to serve them and barriers in accessing and providing SRH services in Rohingya refugee camps in Cox's Bazar, Bangladesh and similar low-and-middle-income country contexts.

Methods and analysis

A mixed-method design will be used in this study. A community-based survey coupled with facility assessments as well as qualitative interviews and discussions will be conducted with community people of Rohingya refugee camps and stakeholders providing SRH services to Rohingya population in Ukhiya and Teknaf upazilas, Cox's Bazar, Bangladesh.

Survey data will be analyzed using univariate, bivariate and multivariable regression statistics. Descriptive analysis will be done for facility assessment and thematic analysis will be conducted with qualitative data.

Ethics and dissemination

Ethical approval from Institutional Review Board of BRAC James P Grant School of Public Health (2018-017-IR) has been obtained. Findings from this research will be disseminated through presentations in local, national and international conferences, workshops, peer-reviewed publications, policy briefs and interactive project report.

Strengths and limitations of this study

- This situation analysis is among the first that will provide an overview of Rohingya women and adolescent girls' (aged 12-59 years) SRH demands and needs, availability and delivery of SRH services, barriers to service uptake and related challenges in Rohingya refugee camps under an acute humanitarian crisis condition
- The study employs a mixed-method approach to assess the current situation, understand the community perspectives and facility readiness to provide different SRH services, related gaps and challenges

For peer review only - http://bmjopen.bmj.com/site/about/guidelines.xhtml

- A methodological limitation of the study is unwillingness of certain respondents (both community level and facility level) to disclose sensitive information related to SRH practices, service utilization and health facility records

Keywords

Sexual and reproductive health, Humanitarian crises, Situation analysis, Rohingya refugees, women, adolescent girls,

Abbreviations used

Sexual and Reproductive Health (SRH), United Nations High Commissioner for Refugees (UNHCR), World Health Organization (WHO), Government of Bangladesh (GoB), United Nations (UN), Traditional Birth Attendants (TBAs), Sustainable Development Goals (SDGs), Inter-Agency Working Group (IAWG), low-and-middle-income countries (LMICs), International non-government organizations (INGOs), National non-government organizations (NNGOs), Focus Group Discussion (FGD), In-depth interview (IDI), Key informant interview (KII)

For peer review only

For peer review only - http://bmjopen.bmj.com/site/about/guidelines.xhtml

INTRODUCTION

To accomplish the target of the 2030 Agenda and the Sustainable Development Goals (SDGs) 3, ensuring healthy lives and promoting well-being for all at all-ages [1] the health needs of refugees and migrants must be corroborated.[2] The world has witnessed a rapid increase in the number of refuges over the past few decades.[3, 4] Refugees are defined as people who are displaced from their homes and cross international borders due to complex emergencies and disasters.[5] According to World Health Organization (WHO) and United Nations High Commissioner for Refugees (UNHCR), globally, total 68.5 million people have been forcibly displaced by the end of 2017 due to political turbulence or natural disasters, persecution, conflict and violence or human rights violations.[4, 6] Estimates from UNHCR (2018) indicate that an estimated 11.8 million people are internally displaced within their own countries, of which 4.4 million are newly displaced refugees. During such humanitarian crises, women, adolescent girls and children comprise more than half of the displaced and refugee population and become the most vulnerable group needing emergency humanitarian response.[7] Being mostly at their reproductive age, women and adolescent girls require access to basic health, safety and well-being needs as well as service delivery including pregnancy, prenatal care, delivery services, postpartum care, family planning services and other reproductive and sexual health related services.[7] Limited or no access to quality sexual and reproductive health (SRH) services during emergency and crisis conditions, put women and adolescent girls at higher risk of morbidity and mortality that requires utmost importance in terms of service design, delivery and implementation.

Muslims in Rakhine state of Western Myanmar have been facing severe humanitarian crisis since the 1982 Citizenship Law that took away their Myanmar citizenship and right to self-identify themselves as Rohingyas.[8] Many Rohingya diaspora thus took shelter in neighboring countries, mostly in Bangladesh due to geographic proximity. Although the Rohingyas have been entering Bangladesh since the 1970s, a large influx happened during 1991-1992.[9] Until August 2017, the number of Rohingya refugees (both registered and unregistered) residing in Cox's Bazar was estimated around 213,000 individuals.[10] An outbreak of violence on Rohingya communities in Myanmar on August 25, 2017 resulted in an influx of more than 700,000 Rohingyas in Cox's Bazar, the southeast coastal district of Bangladesh.[10] This created a grave condition for Bangladesh as a hosting country to immediately respond to the urgent needs of such huge refugee population for food, shelter, clean water, health crises, injuries and traumas with more than half of the population comprising women and adolescent girls.[11]

Responding to this massive influx into Cox's Bazar district of Bangladesh has stretched the capacity of the already over-burdened local administration and health systems.[12] Even though the Government of Bangladesh (GoB), UN agencies, national and international non-government organizations (NGOs) are attempting to respond promptly to the humanitarian crisis for Rohingyas,[12] resolving the crisis needs more integrated contribution from major global players.[10, 12] Furthermore, implementing comprehensive SRH services poses particular challenges in a refugee population not only due to their vulnerability and transitions, but also due to lack of clarity on traditional beliefs and cultural models.[13] Although minimum initial service package of SRH are being rendered by several partner organizations in Rohingya refugee camps of Cox's Bazar,[12] access to essential comprehensive reproductive, maternal and newborn

For peer review only - http://bmjopen.bmj.com/site/about/guidelines.xhtml

health services remains a major concern due to inconsistencies in the quality of services provided, and varying implementation of the established minimum package of health services endorsed by various authorities. Thus, World Health Organization (WHO) is aiming to deliver integrated comprehensive SRH services to meet the immediate SRH needs of extremely vulnerable Rohingya women and adolescent girls who are in acute humanitarian crises, particularly in relation to contraceptive use and safe abortion services. In order to implement such comprehensive SRH service package, a critical first step is to undertake a situation analysis and understand the current state of affairs, cultural and demographic dimensions, and capture lessons learned which is essential for sustainable and successful implementation.[13]

An initial assessment of the current situation in Rohingya refugee camps is pertinent given the acute humanitarian crises and will provide an overview of the needs, availability and delivery of SRH services for adolescent girls and women aged 12-59 years in Rohingya refugee camps. Such exploration can also shed light on the distinctive SRH health needs of Rohingya women and adolescent girls. To explore the facility readiness in providing different SRH services, the gaps in the resources and skills required to provide the comprehensive care, facility level data can also be explored. Thus, a situation analysis in such humanitarian crisis situation will provide a complete understanding of Rohingya women and adolescent girls' SRH demand and needs and barriers to service uptake. Findings from this study will also advance current understanding of implementers like WHO, Inter-Agency Working Group (IAWG) and other key stakeholder on where and how to tailor and improve management and delivery of comprehensive SRH services. This will also allow to explore the possibility of updating and standardizing service and training packages for SRH service providers and drawn upon in future to improve delivery and utilization of comprehensive SRH services in similar humanitarian crises contexts in Bangladesh and other low-and-middle-income countries (LMICs).

STUDY OBJECTIVES

Overall objective of this study is to conduct a situation analysis to assess demand and supply side barriers in accessing SRH services by adolescent girls and women aged 12-59 years in Rohingya refugee camps of Cox's Bazar, Bangladesh.

Specific objectives include:

1. To assess SRH needs and service seeking behavior of Rohingya women and adolescent girls
2. To conduct assessment of facility readiness and explore availability of resources (manpower and essential kits, drugs and supplies), measure gaps and estimation of cost of resources for providing comprehensive SRH services to Rohingya women and adolescent girls
3. To explore demand and supply side challenges in seeking and rendering SRH services
4. To explore scope of improvement of the existent SRHR service delivery system

For peer review only - http://bmjopen.bmj.com/site/about/guidelines.xhtml

METHODS AND ANALYSIS

This study will employ a concurrent mixed methods study design using both qualitative and quantitative techniques. A community-based survey coupled with facility assessments as well as qualitative interviews and discussions will be conducted with a broad range of stakeholders.

Study site

The study will be conducted in Rohingya refugee camps of Ukhiya and Teknaf sub-districts in Cox's Bazar district in the southeast coast of Bangladesh.

Study population

The primary study population is adolescent girls and women (12-59 years) who were forcedly displaced from the Rakhine state of Myanmar and migrated to Bangladesh since August 25, 2017 and residing in the refugee camps of two selected sub-districts of Cox's Bazar district. The secondary study groups include Rohingya males; influential community members; formal and informal healthcare providers; and Government, international/national non-government organizations (INGOs/NNGOs) program staff.

Study design

The following methods will be applied for data collection.

Household survey: A household survey will be conducted among the Rohingya refugee adolescent girls and women (12-59 years) to understand their SRH needs, service utilization, and barriers to access and utilize services. This will enable us determine the gaps in availability and utilization of SRH services.

Qualitative interviews and group discussions: In order to complement the household survey findings, we will also conduct in-depth interviews (IDIs) with adolescent girls and women. This will help to further understand their perspectives about the SRH services available, the challenges they face in accessing and utilizing those services. IDIs with formal and informal healthcare providers who are working in the selected refugee camps will be done to understand the barriers and challenges in providing SRH services to the Rohingya refugee adolescent girls and women. To understand the perspective of their male counterparts, focus group discussions (FGDs) with Rohingya males will also be conducted. Key informant interviews (KIIs) with the key stakeholders from government, INGOs and NNGOs will be conducted to get insights about the existing SRH service delivery system and management challenges. Rohingya community leaders such as Majhees, religious leaders such as Imams and teachers will also be interviewed (KII) to explore their influence on the adolescent girls and women in utilizing SRH services.

Facility assessment: A facility assessment will be undertaken to get an overview about the supply side barriers in terms of infrastructures, human resources including training needs, provision and utilization of SRH services, and medical supplies for serving Rohingya refugee population. This facility assessment exercise will help assess facility readiness to provide comprehensive SRH services. An estimation of resources required for providing comprehensive

For peer review only - http://bmjopen.bmj.com/site/about/guidelines.xhtml

SRH services in the camps will also be done with the data from facility assessment and secondary sources.

Sample size and sampling techniques

The sampling techniques and sample size for each method are described below.

Household survey:

Sample size: Considering available data sources on Rohingya refugees, age and gender breakdown of total refugees were identified from Bangladesh Refugee Emergency Population Fact Sheet.[14] The total number of Rohingya women and adolescent girls aged 12 to 59 years is 269,345 where 60,084 are adolescent girls aged 12 to 17 years and 209,261 are women aged 18 to 59 years. According to UNFPA Monthly Situation Report on Rohingya Humanitarian Response,[15] on May 2018, 22% of pregnant Rohingya women and adolescent girls gave birth in health facilities. Considering this as the prevalence rate with 95% confidence interval, 5% margin of error and 1.5 design effect, the estimated sample size for the household survey is calculated to be 395. Due to large study population (269,345), considering the finite population correction (FPC),[16] the estimated sample size is 395. We considered 10% non-response rate and plan to reach 440 women and adolescent girls.

The formula used for sample size calculation:

$$n = \frac{z^2 p(1-p)}{\mathrm{d}^2} \times \mathrm{deff}$$

Among 269,345 study population, 22.3% (60,084) were adolescent girls aged 12 to 17 years and 77.8% (209,261) were women aged 18 to 59 years. So, the sample has been distributed proportionally among the two groups **(Table 1)**.

Table 1: Distribution of sample size for household survey

Sample population	Number of individuals	Percentage (%)	Sample size considering non-response
Total number of women and adolescent girls aged 12 to 59 years	269,345	100%	440
Adolescent girls aged 12 to 17 years	60,084	22.3%	99
Women aged 18 to 59 years	209,261	77.7%	341

Sampling Techniques: A multistage sampling approach will be employed for selecting camps and study respondents **(Figure 1)**.

Stage I: In the first stage sampling, eleven camps will be randomly selected from 34 camps; that represents 30% of the total camps in Ukhiya and Teknaf sub-districts at Cox's Bazar. Refugee camps in Ukhiya and Teknaf sub-districts where new makeshifts were established after the August 25, 2017 influx will be selected. Registered refugee camps with old settlements where Rohingya refugees are living since before the influx of August 25, 2017 will be excluded. The

For peer review only - http://bmjopen.bmj.com/site/about/guidelines.xhtml

samples will be equally distributed among the 11 selected camps (40 sample per camp). Survey data will be collected from 31 women and 9 adolescent girls aged 12-17 years from each camp considering proportionality.

Stage II: In 2nd stage sampling, a complete list of Majhiis (local community leaders of Rohingyas) of the selected 11 camps will be collected from the Camp in-Charge (CiC) office and one Majhii will be randomly selected per camp.

Stage III: The house of the selected Majhii will be determined as starting point to select the sample households in third stage. Selected from both left and right side, the 1st (closest) sample household will be after 10 footsteps from Majhii's house. Accordingly, households in every 10 footsteps will be selected until desired number of respondents are interviewed. One married woman will be picked from every selected household as a respondent. If more than one married woman is found in a household, then one will be randomly selected on the basis of availability and interest for interviewing. In addition, if available, an adolescent (aged 12-17) will be interviewed from the same household. If more than one adolescent girls in that household, then same procedure will be followed. If no adolescent girls are found in any sample household, then the third stage will be repeated till required number of adolescent girls are surveyed.

Qualitative data: For qualitative interviews, three camps will be selected from eleven camps (where household survey and facility assessment will be conducted) depending on geographic location, challenging terrains, remoteness, difficulty in accessibility, availability of infrastructure etc. The sampling strategy, and type and number of respondents for each of the qualitative activities planned is provided in **Table 2**.

Table 2. Sampling frame and characteristics for each qualitative activity

Activity & Focus	Sampling Strategy	Respondent Groups	Estimated Numbers
Activity 1: KIIs	Opportunistic/ emergent sampling<br><br>Snowball sampling	Local and international NGO programme leads, managers, SRH focal points, influential workers, government high officials, programme managers	5
		Influential community members: Majhii, imam, lady imam, Burmese teachers	9
Activity 2: IDIs	Purposive sampling	Rohingya women aged 18-59 years	9
		Rohingya adolescent girls (12-17 years old)	9
	Opportunistic/ emergent sampling	Formal providers such as midwife, health center in-charge, doctors, nurses, community health workers	11
		Informal providers such as traditional birth attendants (TBAs), Burmese doctor, traditional healers,	9
Activity 3: FGDs	Purposive sampling	Rohingya males	3

For peer review only - http://bmjopen.bmj.com/site/about/guidelines.xhtml

Facility Assessment: For facility assessment, five categories of health facilities will be chosen according to WHO Health Facility Register (shared internally by WHO). The categories include: primary health centers (PHC), health posts (fixed and plus), labor rooms or SRH only facilities, secondary health facility and community clinics. One facility from each category will be randomly selected for assessment. Facilities functional during data collection will be considered for random sampling. PHCs, health posts plus and fixed health post, and labor rooms/ SRH only facilities/maternity centers are camp-specific and situated inside the camp. Secondary hospitals and community clinics are situated outside the camps. If only one facility is found in a selected category, then that facility will be assessed. Where more than one facility is available in a category, one facility will be selected randomly for this study. For secondary health facilities, based on proximity to camp locations random selection will be conducted. In both sub-districts, two government secondary facilities (Upazila Health Complex) are serving as the main referral points. Additionally, 2 community clinics are GoB-run primary level facilities under Ministry of Health and Family Welfare. So, all of these health facilities will be selected for assessment. Therefore, we will be conducting facility assessment in total 29 health facilities - 11 health posts (fixed), 9 PHCs, 2 labor rooms and SRH only facilities, 5 secondary health facilities and 2 community clinics altogether.

Data Collection methods and tools

Various tools will be used for collecting data by different methods. All tools will be finalized after pre-testing in similar camps that are not selected for this study.

Household survey: A structured quantitative questionnaire will be prepared following a guideline prepared by UNFPA and Save the Children for humanitarian crisis situation[17] and scholarly literatures.[18] Data will be collected through Samsung tablets (Model no. SM-T231) by using SurveyCTO software, an Open Data Kit (ODK) tool widely used for collecting survey data. Using ODK will ensure automatic data storage in database which will be converted to statistical software package Stata 13 (StataCorp, 4905 Lakeway Drive, College Station, Texas, USA) for data cleaning and analyzing. The questionnaire will be translated in Bangla from English including key SRHR terms in Rohingya language. Local dialects will be used in the questions for clarity of our study objective related topics (local language). Local experienced female interviewers, who understand the language of Rohingya community, will be recruited for data collection. Extensive training sessions will be conducted to orient and train them regarding the study objective and tools. The interviewers will be monitored by two researchers and one statistician of BRAC JPGSPH while collecting data. In addition, two local male interviewers will be recruited for building rapport with the community key persons in each camp.

Qualitative data: Separate guidelines will be developed for IDIs and KIIs with different groups and FGDs with males. Qualitative interviews will be conducted by an experienced group of researchers trained in qualitative interviewing and analysis. However, due to language barrier, interpreters will be recruited from the nearby locality who understand the language and dialect of Rohingya community. Training sessions will be conducted to orient and train them on study objective and qualitative tools prior interviewing. A period of rapport building with the community key persons in each camp site will be critical to the success of this research given known difficulties in accessing the Rohingya population, their conservative cultures, suspicion about motives and post-interview repercussions. Interviews with women and adolescent girls will only be conducted by female researchers and interpreters due to conservative nature of the local

For peer review only - http://bmjopen.bmj.com/site/about/guidelines.xhtml

population and nature of the questions involved. These dynamics must be handled carefully, or else access will be hampered. Networks with influential and key locals will be important in opening doors and initiating discussion. Male researchers including local male interpreters will conduct FGDs, IDIs and KIIs with community males.

Facility assessment: A structured English questionnaire will be prepared for facility assessment following WHO Service Availability and Readiness Assessment (SARA) tool [15]. Two researchers will collect data from different categories of health facilities identified. Data will be collected through Samsung tablets (Model no. SM-T231) by using KOBO software, an ODK tool.

Data Analysis

Household survey: Descriptive analyses will be performed on survey data collected to understand socio demographic characteristics, need for SRH services, health care and service seeking behavior, service utilization patterns and barriers to accessing services, challenges faced on the basis of distance and waiting time at health facilities, their restrictions and reasons for not taking services etc. Analysis will be performed in separate groups for women (18- 59 years) and adolescent girls (12-17 years) to understand their specific SRH needs. Depending on the distribution of variables, frequencies, percentages, mean (standard deviation) and range as summary statistics will be reported. Chi-square test will be performed to measure the association between socio-demographic characteristics and other variables of menstrual health, pregnancy, delivery care, family planning services, menstrual regulation, abortion and sexual transmitted diseases, and feasible challenges of demand side and pattern of health seeking behavior for sexual and reproductive healthcare. Multivariable regression analysis will be conducted afterwards if significant associations are found in bivariate analyses.

Qualitative data: An outline of the plan for qualitative data analysis will be prepared in advance of the research which will include defining a priori codes according to study objectives. All interviews will be recorded provided consent has been obtained, along with simultaneous note-taking in case of equipment failure. Data transcription will occur immediately following each interview, followed by translation. Data familiarization will involve reading transcripts repeatedly to surface emerging themes, assess strengths and weaknesses of interview techniques, and identify any missed opportunities for further exploration. Transcripts will be reviewed carefully, and coding will be done following the a priori code-list. A team approach to analysis will be employed to minimize individual biases. Intra-coder and inter-coder reliability will be checked. This approach is applied in all aspects of analysis including coding, with multiple analysts coding the same sections of text to assess inter-coder reliability. Group discussions of emerging themes and patterns in the data will be tested using data displays that allow more systematic pattern-testing across respondents. Any emerging codes identified during analysis will be added in the code-list after confirmation as a team and will be used for coding all transcripts.

Facility assessment: Descriptive analysis will be conducted according to the type of facilities to understand the facility readiness and challenges faced by supply side. The analysis will be performed separately for five different categories proposed to identify gaps at all levels - service provision and availability, service utilization, human resources including their training,

For peer review only - http://bmjopen.bmj.com/site/about/guidelines.xhtml

infrastructure, and supply of equipment and drugs for providing SRH services by the health facilities.

ETHICS AND DISSEMINATION

This study has sought ethical approval from Institutional Review Board (IRB) of BRAC James P Grant School of Public Health (2018-017-IR) and poses no more than minimal risk to subjects. Respondents will be asked for written consent prior interviewing. Written ascent will be taken from adolescent respondents and written consents will be sought from their guardians.

Tape recorders will be used for recording the qualitative interviews in order to collect full and intact thoughts. Strong password-protected server or user profile will be created and utilized for quantitative data collection using Open Data Kit (ODK) tools - SurveyCTO for survey and KoBo Toolbox for facility assessment. All forms of data related to this study will be stored in locked storage or controlled-access folders allowing access by authorized persons related to the study i.e. Principal Investigator, other study investigators, and IRB members of BRAC JPGSPH.

Findings from this research will be disseminated at various levels so that evidence generated can be advocated and translated into policy actions for better SRH of Rohingya refugee women and adolescent girls. Findings will be presented to relevant local administrators, development partners and NGOs and other relevant parties, academicians and researchers through local and national conferences, dissemination workshops, interactive project report and policy briefs. Additionally, scholarly publications in peer-reviewed journals and presentations in international scientific forums, conferences, and symposiums will be done for international audiences.

EXPECTED CHALLENGES

First and foremost, the challenge expected by researchers of this study is the language barrier as Rohingya people cannot speak or understand Bengali or English languages. Hence, it will be difficult to understand their dialect for the researchers unless trained interpreters are involved. Thus, we will recruit data collectors cum interpreters from the local Bangladeshi community who can speak and understand Rohingya language fluently. They will act as key persons to establish communication between researchers and Rohingya people. However, a list of key terms in Rohingya language and translation into Bangla and English will be prepared with the help of data collectors as a reference for the researchers who will be accompanying the local data collectors to combine their critical thinking with the community dialect. They will also perform spot-checks while collecting data. Another challenge is the sensitive nature of the questions to be asked in this study given the conservative culture of the Rohingya population. Only female data collectors and researchers will interview female respondents and male data collectors and researchers will conduct interviews and FGDs with male respondents. Data collectors will be trained to respect the current humanitarian crises condition in the Rohingya camps, the trauma this community has experienced, their culture, choices and privacy of the interview respondents. Another major challenge can be the entry into local Rohingya community. Harnessing the power

For peer review only - http://bmjopen.bmj.com/site/about/guidelines.xhtml

relations and dynamics in the Rohingya community is the key to conduct such situation analysis. Thus, the study is designed accordingly to draw on the benefits of engaging gatekeepers to create access in the community for data collection. After entering in each camp, the researchers will communicate with the selected Majhiis, the local Rohingya leaders, and explain him/her the purpose of the study. After leveraging sufficient time to build rapport with the Majhiis, their support will be sought to get access to the households. In terms of working in facilities, accessing the records could be difficult, hence, the higher authority of each facility will be informed beforehand. Another challenge in this study can be political unrest like strike for which data collection may delay. Finally, weather and geographical difficulties is one of the biggest challenges in working at Rohingya camps as located in hilly areas and challenging terrains. In order to avoid any sort of accident, adequate logistics support to the data collectors and study researchers will be ensured.

For peer review only

For peer review only - http://bmjopen.bmj.com/site/about/guidelines.xhtml

Contributors

RA, NF, BA, RH, SBS, PR, AA, AR, MTH, ZQ, SFR contributed in conceptualization or design of the study and VU, LHK, JR, LS reviewed and incorporated their critical inputs. RH, SBS and PR have contributed equally. Also, VU, LHK, JR and LS made equal contributions. RA drafted the initial version with support from NF and BA. MTH, ZQ, SFR reviewed and helped revise critically. RH, SBS, AA, PR, AR, VU, LHK, JR, LS reviewed critically for important intellectual content. RA finally revised the version submitted with inputs from all other co-authors. All authors finally approved the version published. RA, BA, MTH, ZQ, SFR, LHK are in agreement to be accountable for all aspects of the work in ensuring that questions related to the accuracy or integrity of any part of the work are appropriately investigated and resolved.

Competing interests

The authors declare no competing interests.

Funding

Funding is received from World Health Organization (WHO) for this work; Grant Number: 201991701 and Unit Reference - FWC/RHR

Patient consent

Not required

Ethics approval

Institutional Review Board (IRB) of BRAC James P Grant School of Public Health.

For peer review only

For peer review only - http://bmjopen.bmj.com/site/about/guidelines.xhtml

REFERENCES

1. Sustainable Development Goals. Goal 3: Ensure healthy lives and promote well-being for all at all ages. United Nations. Available from: https://www.un.org/sustainabledevelopment/health/
2. World Health Organization. Refugee and migrant health. *Reports on situation analysis and practices in addressing the health needs of refugees and migrants.* Available from: http://www.who.int/migrants/publications/situation-analysis-reports/en/
3. Robinson V (Ed.). The international refugee crisis: British and Canadian responses: Springer; 2016. Palgrave Macmillan; 1st ed. 1993, edition: 2016. DOI: 10.1007/978-1-349-12054-3
4. United Nations High Commissioner for Refugees (UNHCR). *Global Trends: Forced Displacement in 2017.* 2018 June 25. Available from: http://www.unhcr.org/statistics/unhcrstats/5b27be547/unhcr-global-trends-2017.html [Accessed on July 30, 2018].
5. World Health Organization. Environmental health in emergencies. Displaced people. https://www.who.int/environmental_health_emergencies/displaced_people/en/ [Accessed on July 31, 2018]
6. World Health Organization. *2015 WHO Humanitarian Response.* Department for Emergency Risk Management and Humanitarian Response (ERM). 2015. Available from: http://www.who.int/hac/who_humanitarian_response_plans2015.pdf [Accessed on July 30, 2018].
7. United Nations Population Fund (UNFPA). *Protecting Women in Emergency Situations.* Available from: https://www.unfpa.org/resources/protecting-women-emergency-situations [Accessed on August 1, 2018].
8. United Nations Office for the Coordination of Humanitarian Affairs (OCHA U). *Cluster coordination.* Available from: http://www.unocha.org/what-we-do/coordination-tools/cluster-coordination. [Accessed July 30, 2018].
9. Haque MM. Rohingya Ethnic Muslim Minority and the 1982 Citizenship Law in Burma. *Journal of Muslim Minority Affairs* 2017;37(4):454-69.
10. Khatun F. *Implications of the Rohingya Crisis for Bangladesh.* Centre for Policy Dialogue (CPD): Dhaka, Bangladesh, 2017 November 11.
11. International Organization for Migration (IOM). *Needs and Population Monitoring (NPM). Site Assessment: Round 10; 2018.* May 20, 2018.
12. *Rohingya Crisis in Cox's Bazar, Bangladesh Health Sector Bulletin.* Health Sector Coordination teamApril 12, 2018. Available from: http://www.searo.who.int/bangladesh/healthsectorcxbbanbulletinno4.pdf?ua=1 [Accessed on July 30, 2018].
13. Doedens W, Burns K. *Challenges to reproductive health in emergencies.* Health in Emergencies. Department of Emergency and Humanitarian Action. World Health Organization (WHO). Issue 10, June 2001;P 1-7.
14. Reliefweb. *Bangladesh Refugee Emergency Population Factsheet* (as of 30 June, 2018). Available from: https://reliefweb.int/sites/reliefweb.int/files/resources/64651.pdf
15. United Nations Population Fund Bangladesh (UNFPA). *UNFPA Rohingya Humanitarian Response. Monthly Situation Report.* May 2018. Available from:

For peer review only

For peer review only - http://bmjopen.bmj.com/site/about/guidelines.xhtml

https://bangladesh.unfpa.org/sites/default/files/pub-pdf/UNFPA_SitRep_External__17%20May%202018%20Final.pdf
16. Cochran WG. Sampling techniques. John Wiley & Sons. 2007;Pg. 24. Available from: http://krishikosh.egranth.ac.in/bitstream/1/2034365/1/CIFE-100.pdf
17. UNFPA and Save the Children. *Adolescent Sexual and Toolkit for Reproductive Health Humanitarian Settings: A Companion to the Inter-Agency Field Manual on Reproductive Health in Humanitarian Settings*. 2009. 99 p.
18. Pyone T, Dickinson F, Kerr R, et al. Data collection tools for maternal and child health in humanitarian emergencies: a systematic review. *Bull World Health Organ* 2015;93(9):648–658A. Available from: http://www.who.int/entity/bulletin/volumes/93/9/14-148429.pdf

For peer review only

For peer review only - http://bmjopen.bmj.com/site/about/guidelines.xhtml

Figure 1: Sampling techniques to conduct situation analysis

For peer review only

For peer review only - http://bmjopen.bmj.com/site/about/guidelines.xhtml

Figure 1: Sampling techniques to conduct situation analysis

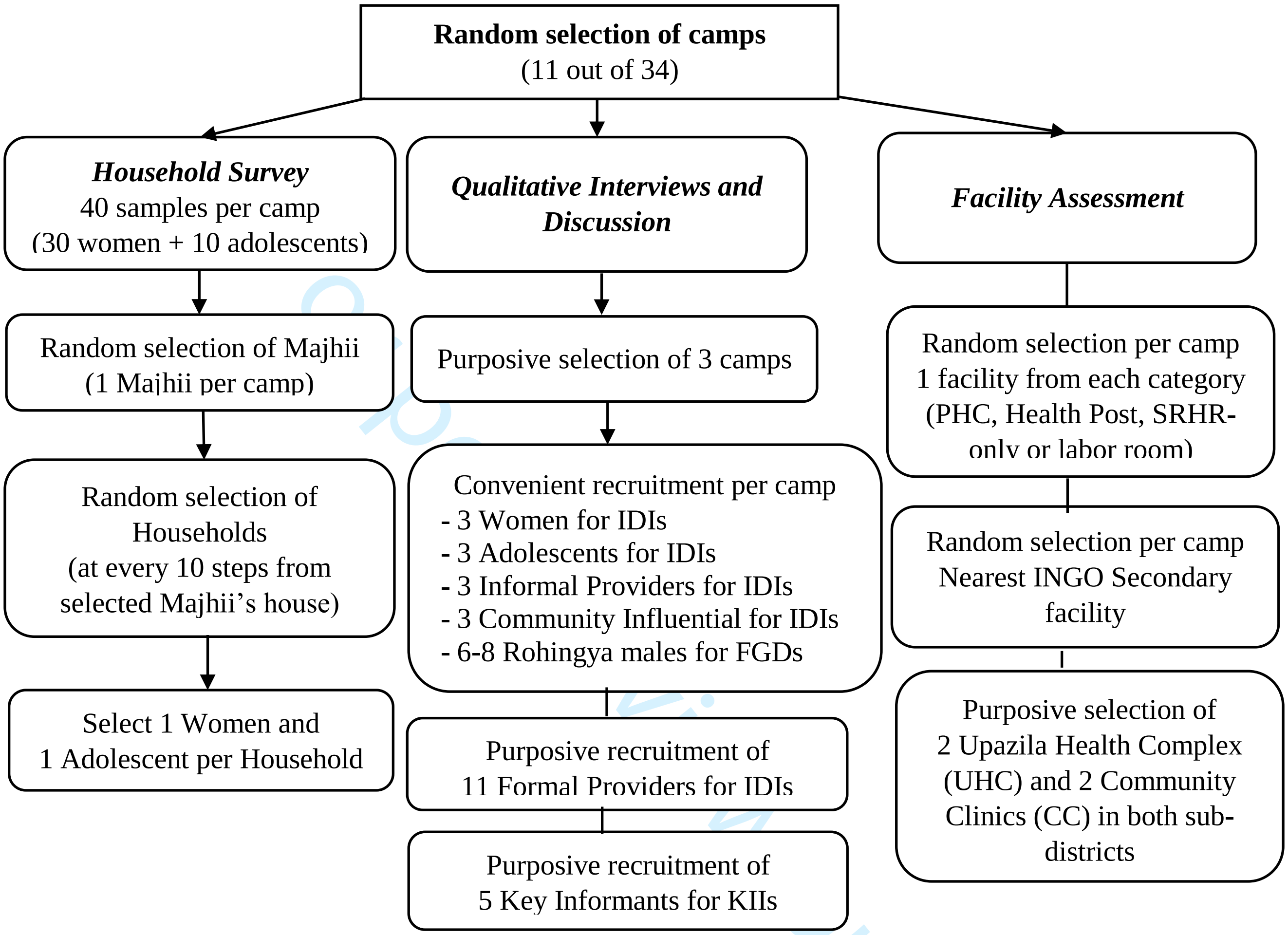

STROBE 2007 (v4) Statement—Checklist of items that should be included in reports of *cross-sectional studies*

Section/Topic	Item #	Recommendation	Reported on page #
**Title and abstract**	1	(*a*) Indicate the study's design with a commonly used term in the title or the abstract	1, 2
		(*b*) Provide in the abstract an informative and balanced summary of what was done and what was found	2
**Introduction**			
Background/rationale	2	Explain the scientific background and rationale for the investigation being reported	4, 5
Objectives	3	State specific objectives, including any prespecified hypotheses	5
**Methods**			
Study design	4	Present key elements of study design early in the paper	6
Setting	5	Describe the setting, locations, and relevant dates, including periods of recruitment, exposure, follow-up, and data collection	6, 7, 8, 9
Participants	6	(*a*) Give the eligibility criteria, and the sources and methods of selection of participants	6, 7, 8, 9
Variables	7	Clearly define all outcomes, exposures, predictors, potential confounders, and effect modifiers. Give diagnostic criteria, if applicable	10
Data sources/ measurement	8*	For each variable of interest, give sources of data and details of methods of assessment (measurement). Describe comparability of assessment methods if there is more than one group	N/A
Bias	9	Describe any efforts to address potential sources of bias	10
Study size	10	Explain how the study size was arrived at	7, 8, 9
Quantitative variables	11	Explain how quantitative variables were handled in the analyses. If applicable, describe which groupings were chosen and why	10
Statistical methods	12	(*a*) Describe all statistical methods, including those used to control for confounding	10
		(*b*) Describe any methods used to examine subgroups and interactions	10
		(*c*) Explain how missing data were addressed	N/A
		(*d*) If applicable, describe analytical methods taking account of sampling strategy	7
		(*e*) Describe any sensitivity analyses	N/A
**Results**			

For peer review only

Participants	13*	(a) Report numbers of individuals at each stage of study—eg numbers potentially eligible, examined for eligibility, confirmed eligible, included in the study, completing follow-up, and analysed	N/A
		(b) Give reasons for non-participation at each stage	N/A
		(c) Consider use of a flow diagram	N/A
Descriptive data	14*	(a) Give characteristics of study participants (eg demographic, clinical, social) and information on exposures and potential confounders	N/A
		(b) Indicate number of participants with missing data for each variable of interest	N/A
Outcome data	15*	Report numbers of outcome events or summary measures	N/A
Main results	16	(*a*) Give unadjusted estimates and, if applicable, confounder-adjusted estimates and their precision (eg, 95% confidence interval). Make clear which confounders were adjusted for and why they were included	N/A
		(*b*) Report category boundaries when continuous variables were categorized	N/A
		(*c*) If relevant, consider translating estimates of relative risk into absolute risk for a meaningful time period	N/A
Other analyses	17	Report other analyses done—eg analyses of subgroups and interactions, and sensitivity analyses	N/A
**Discussion**			
Key results	18	Summarise key results with reference to study objectives	N/A
Limitations	19	Discuss limitations of the study, taking into account sources of potential bias or imprecision. Discuss both direction and magnitude of any potential bias	2, 3, 11, 12
Interpretation	20	Give a cautious overall interpretation of results considering objectives, limitations, multiplicity of analyses, results from similar studies, and other relevant evidence	N/A
Generalisability	21	Discuss the generalisability (external validity) of the study results	N/A
**Other information**			N/A
Funding	22	Give the source of funding and the role of the funders for the present study and, if applicable, for the original study on which the present article is based	13

*Give information separately for cases and controls in case-control studies and, if applicable, for exposed and unexposed groups in cohort and cross-sectional studies.

Note: An Explanation and Elaboration article discusses each checklist item and gives methodological background and published examples of transparent reporting. The STROBE checklist is best used in conjunction with this article (freely available on the Web sites of PLoS Medicine at http://www.plosmedicine.org/, Annals of Internal Medicine at http://www.annals.org/, and Epidemiology at http://www.epidem.com/). Information on the STROBE Initiative is available at www.strobe-statement.org.

For peer review only

BMJ Open

Situation analysis for delivering integrated comprehensive sexual and reproductive health services in humanitarian crisis condition for Rohingya refugees in Cox's Bazar, Bangladesh: protocol for a mixed-method study

Journal:	*BMJ Open*
Manuscript ID	bmjopen-2018-028340.R1
Article Type:	Protocol
Date Submitted by the Author:	08-Mar-2019

Complete List of Authors:	AHMED, RUSHDIA; BRAC University James P Grant School of Public Health<br>Farnaz, Nadia; BRAC University James P Grant School of Public Health<br>Aktar, Bachera; BRAC University James P Grant School of Public Health<br>Hassan, Raafat; BRAC University James P Grant School of Public Health<br>Shafique , Sharid ; BRAC University James P Grant School of Public Health<br>Ray, Pushpita ; BRAC University James P Grant School of Public Health<br>Awal, Abdul ; BRAC University James P Grant School of Public Health<br>Rahman, Atiya; BRAC University James P Grant School of Public Health<br>Urbaniak , Veronique; World Health Organization , Reproductive Health and Research<br>Kobeissi, Loulou; World Health Organization , Reproductive Health and Research<br>Rosie, Jeffries; World Health Organization , Health Sector Coordination Office<br>Say, Lale; World Health Organization, Reproductive Health and Research<br>Hasan, Md Tanvir; BRAC University James P Grant School of Public Health<br>Quayyum , Zahidul ; BRAC University James P Grant School of Public Health<br>Rashid, Sabina Faiz; BRAC University James P Grant School of Public Health

**Primary Subject Heading**:	Public health
Secondary Subject Heading:	Sexual health
Keywords:	Sexual and reproductive health, humanitarian crises, Situation analysis, Rohingya refugees, Women, Adolescent girls

SCHOLARONE™
Manuscripts

For peer review only - http://bmjopen.bmj.com/site/about/guidelines.xhtml

Situation analysis for delivering integrated comprehensive sexual and reproductive health services in humanitarian crisis condition for Rohingya refugees in Cox's Bazar, Bangladesh: protocol for a mixed-method study

Rushdia Ahmed[1], Nadia Farnaz[1], Bachera Aktar[1], Raafat Hassan[1], Sharid Bin Shafique[1], Pushpita Ray[1], Abdul Awal[1], Atiya Rahman[1], Veronique Urbaniak[2], Loulou Hassan Kobeissi[2], Jeffries Rosie[3], Lale Say[2], Md Tanvir Hasan[1], Zahidul Quayyum[1], Sabina Faiz Rashid[1]

[1] BRAC James P Grant School of Public Health, BRAC University, Dhaka, Bangladesh
[2] Department of Reproductive Health Research, World Health Organization, Geneva, Switzerland
[3] Health Sector Coordination Office, World Health Organization, Cox's Bazar, Bangladesh

*Corresponding author: Rushdia Ahmed (RA)

Contact Address: 68, Shaheed Tajuddin Ahmed Sarani, Mohakhali, Dhaka, Bangladesh – 1212. Telephone number: +880 178 174 2055.

Email: ahmed.rushdia@yahoo.com

For peer review only

For peer review only - http://bmjopen.bmj.com/site/about/guidelines.xhtml

ABSTRACT

Introduction

Rohingya diaspora are one of the most vulnerable groups seeking refuge in camps of Cox's Bazar, Bangladesh, arising an acute humanitarian crisis. More than half of the Rohingya refugees are women and adolescent girls requiring quality sexual and reproductive health (SRH) services. Minimum initial service package of SRH are being rendered in the refugee camps, however, World Health Organization is aiming to provide integrated comprehensive SRH services to meet the unmet needs of this most vulnerable group. For sustainable and successful implementation of such comprehensive SRH service packages, a critical first step is to undertake a situation analysis and understand the current dimensions, and capture the lessons learned on their SRH specific needs and implementation challenges. This situation analysis is pertinent in current humanitarian condition and will provide an overview of the needs, availability and delivery of SRH services for adolescent girls and women, barriers in accessing and providing those services in Rohingya refugee camps in Cox's Bazar, Bangladesh and similar humanitarian contexts.

Methods and analysis

A concurrent mixed-methods design will be used in this study. A community-based household survey coupled with facility assessments as well as qualitative in-depth interviews, key informant interviews and focus group discussions will be conducted with community people of Rohingya refugee camps and relevant stakeholders providing SRH services to Rohingya population in Cox's Bazar, Bangladesh.

Survey data will be analyzed using univariate, bivariate and multivariable regression statistics. Descriptive analysis will be done for facility assessment and thematic analysis will be conducted with qualitative data.

Ethics and dissemination

Ethical approval from Institutional Review Board of BRAC James P Grant School of Public Health (2018-017-IR) has been obtained. Findings from this research will be disseminated through presentations in local, national and international conferences, workshops, peer-reviewed publications, policy briefs and interactive project report.

Strengths and limitations of this study

- This situation analysis is among the first that will provide an overview of Rohingya women and adolescent girls' (aged 12-59 years) SRH demands and needs, availability and delivery of SRH services, barriers to service uptake and related challenges in Rohingya refugee camps under an acute humanitarian crisis condition
- The study will utilize a concurrent mixed-methods design to assess the current situation, understand the community perspectives and facility readiness to provide different SRH services, related gaps and challenges

For peer review only - http://bmjopen.bmj.com/site/about/guidelines.xhtml

- While designing the study, sampling was done using facility delivery rate available in the existing literature body as no prevalence data on other SRH indicators such as family planning, abortion, or menstruation were found
- A potential limitation foreseen within this study is the unwillingness of certain respondents (both community level and facility level) to disclose sensitive information related to SRH practices, service utilization and health facility records

Keywords

Sexual and reproductive health, Humanitarian crises, Situation analysis, Rohingya refugees, women, adolescent girls,

Abbreviations used

Sexual and Reproductive Health (SRH), United Nations High Commissioner for Refugees (UNHCR), World Health Organization (WHO), Government of Bangladesh (GoB), United Nations (UN), Traditional Birth Attendants (TBAs), Sustainable Development Goal (SDG), Inter-Agency Working Group (IAWG), low-and-middle-income countries (LMICs), International non-government organizations (INGOs), National non-government organizations (NNGOs), Focus Group Discussion (FGD), In-depth interview (IDI), Key informant interview (KII)

For peer review only

INTRODUCTION

To accomplish the target of the 2030 Agenda and the Sustainable Development Goal (SDG) 3, ensuring healthy lives and promoting well-being for all at all-ages,[1] the health needs of refugees and migrants must be corroborated.[2] The world has witnessed a rapid increase in the number of refuges over the past few decades.[3, 4] Refugees are defined as people who are displaced from their homes and cross international borders due to complex emergencies and disasters.[5] According to World Health Organization (WHO) and United Nations High Commissioner for Refugees (UNHCR), globally, total 68.5 million people have been forcibly displaced by the end of 2017 due to political turbulence or natural disasters, persecution, conflict and violence or human rights violations.[4, 6] Estimates from UNHCR (2018) indicate that an estimated 11.8 million people are internally displaced within their own countries, of which 4.4 million are newly displaced persons. During such humanitarian crises, women, adolescent girls and children comprise more than half of the displaced and refugee population and become the most vulnerable groups needing emergency humanitarian response.[7] Being mostly at their reproductive age, women and adolescent girls require access to basic health, safety and well-being needs as well as service delivery including pregnancy, prenatal care, delivery services, postpartum care, family planning services and other reproductive and sexual health related services.[7] Limited or no access to quality sexual and reproductive health (SRH) services during emergency and crisis conditions, put women and adolescent girls at higher risk of morbidity and mortality that requires utmost importance in terms of service design, delivery and implementation.

Muslims in Rakhine state of Western Myanmar have been facing severe humanitarian crisis since the 1982 Citizenship Law that took away their Myanmar citizenship and right to self-identify themselves as Rohingyas.[8] Many Rohingya diaspora thus took shelter in neighboring countries, mostly in Bangladesh due to geographic proximity. Although the Rohingyas have been entering Bangladesh since the 1970s, a large influx happened during 1991-1992.[9] Until August 2017, the number of Rohingya refugees (both registered and unregistered) residing in Cox's Bazar was estimated around 213,000 individuals.[10] An outbreak of violence on Rohingya communities in Myanmar on August 25, 2017 resulted in an influx of more than 700,000 Rohingyas in Cox's Bazar, the southeast coastal district of Bangladesh.[10] This created a grave condition for Bangladesh as a hosting country to immediately respond to the urgent needs of such huge refugee population for food, shelter, clean water, health crises, injuries and traumas with more than half of the population comprising women and adolescent girls.[10, 11]

Responding to this massive influx into Cox's Bazar district of Bangladesh has stretched the capacity of the already over-burdened local administration and health systems.[11] Even though the Government of Bangladesh (GoB), UN agencies, national and international non-government organizations (NGOs) are attempting to respond promptly to the humanitarian crisis for Rohingyas,[11] resolving the crisis needs more integrated contribution from major global players.[11] Furthermore, implementing comprehensive SRH services poses particular challenges in a refugee population not only due to their vulnerability and transitions, but also due to lack of clarity on traditional beliefs and cultural models.[12] Although minimum initial service package of SRH (i.e. priority set of lifesaving activities to respond to reproductive health needs at the onset of humanitarian crisis condition) are being rendered by several partner organizations

For peer review only - http://bmjopen.bmj.com/site/about/guidelines.xhtml

in Rohingya refugee camps of Cox's Bazar,[11] access to essential comprehensive reproductive, maternal and newborn health services remains a major concern due to inconsistencies in the quality of services provided, and varying implementation of the established minimum package of health services endorsed by various authorities. Thus, World Health Organization (WHO) is aiming to deliver integrated comprehensive SRH services to meet the immediate SRH needs of extremely vulnerable Rohingya women and adolescent girls who are in acute humanitarian crises. In order to implement such comprehensive SRH service package, a critical first step is to undertake a situation analysis and understand the current state of affairs, cultural and demographic dimensions, and capture lessons learned which is essential for sustainable and successful implementation.[12]

An initial assessment of the current situation in Rohingya refugee camps is pertinent given the acute humanitarian crises and will provide an overview of the needs, availability and delivery of SRH services for adolescent girls and women aged 12-59 years in Rohingya refugee camps. Such exploration can also shed light on the distinctive SRH health needs of Rohingya women and adolescent girls. To explore the facility readiness in providing different SRH services, the gaps in the resources and skills required to provide the comprehensive care, facility level data can also be explored. Thus, a situation analysis in such humanitarian crisis situation will provide a complete understanding of Rohingya women and adolescent girls' SRH demand and needs and barriers to service uptake. Findings from this study will also advance current understanding of implementers like WHO, Inter-Agency Working Group (IAWG) and other key stakeholder on where and how to tailor and improve management and delivery of comprehensive SRH services. This will also allow to explore the possibility of updating and standardizing service and training packages for SRH service providers and drawn upon in future to improve delivery and utilization of comprehensive SRH services in similar humanitarian crises contexts in Bangladesh and other low-and-middle-income countries (LMICs).

STUDY OBJECTIVES

Overall objective of this study is to conduct a situation analysis to assess demand and supply side barriers in accessing SRH services by adolescent girls and women aged 12-59 years in Rohingya refugee camps of Cox's Bazar, Bangladesh.

Specific objectives include:

1. To assess SRH needs and service seeking behavior of Rohingya women and adolescent girls
2. To conduct assessment of facility readiness and explore availability of resources (manpower and essential kits, drugs and supplies), measure gaps and estimation of cost of resources for providing comprehensive SRH services to Rohingya women and adolescent girls
3. To explore demand and supply side challenges in seeking and rendering SRH services
4. To explore scope of improvement of the existent SRHR service delivery system

For peer review only - http://bmjopen.bmj.com/site/about/guidelines.xhtml

METHODS AND ANALYSIS

Study design and population

This study will employ a concurrent mixed methods study design using both qualitative and quantitative techniques. A community-based survey coupled with facility assessments as well as qualitative in-depth interviews (IDIs), key informant interviews (KIIs) and focus group discussions (FGDs) will be conducted with a broad range of stakeholders. The primary study population is adolescent girls and women (12-59 years) who were forcedly displaced from the Rakhine state of Myanmar and migrated to Bangladesh since August 25, 2017 and residing in the refugee camps of two selected sub-districts of Cox's Bazar district. The secondary study groups include Rohingya males; influential community members; formal and informal healthcare providers; and Government, international/national non-government organizations (INGOs/NNGOs) program staff. Duration of the study is 1 year starting from July 20, 2018.

Study site

The study will be conducted in Rohingya refugee camps of Ukhiya and Teknaf sub-districts in Cox's Bazar district in the southeast coast of Bangladesh. In total, 34 refugee camps are located in these sites as per Health Sector, Cox's Bazar, June 2018 data, where only two are registered and pre-existing settlements. The congested camp environment along with fragile forest, hilly terrain induced geographical difficulty and seasonal variation including monsoon and rainfall, make the life of Rohingya diaspora critical.

Data collection

The following methods will be applied for data collection **(Figure 1)**.

Household survey*:* A household survey will be conducted among the Rohingya refugee adolescent girls and women (12-59 years) to understand their SRH needs, service utilization, and barriers to access and utilize services. The survey will capture information related to health and care seeking of Rohingya women and adolescent girls, especially on menstrual health, pregnancy and delivery care, postnatal care, family planning services utilized, menstrual regulation (MR) and abortion, sexually transmitted diseases, service utilization and barriers related to accessing services. This will enable us determine the gaps in availability and utilization of SRH services.

<u>Sample size and sampling techniques:</u>

Sample size: Considering available data sources on Rohingya refugees, age and gender breakdown of total refugees were identified from Bangladesh Refugee Emergency Population Fact Sheet.[13] The total number of Rohingya women and adolescent girls aged 12 to 59 years is 269,345 where 60,084 are adolescent girls aged 12 to 17 years and 209,261 are women aged 18 to 59 years. According to UNFPA Monthly Situation Report on Rohingya Humanitarian Response,[14] on May 2018, 22% of pregnant Rohingya women and adolescent girls gave birth in health facilities. Considering this as the prevalence rate with 95% confidence interval, 5% margin of error and 1.5 design effect, the estimated sample size for the household survey is calculated to be 395. Due to large study population (269,345), considering the finite population

For peer review only - http://bmjopen.bmj.com/site/about/guidelines.xhtml

correction (FPC),[15] the estimated sample size is 395. We considered 10% non-response rate and plan to reach 440 women and adolescent girls.

The formula used for sample size calculation:

$$n = \frac{z^2 p(1-p)}{d^2} \times \text{deff}$$

Among 269,345 study population, 22.3% (60,084) were adolescent girls aged 12 to 17 years and 77.7% (209,261) were women aged 18 to 59 years. So, the sample has been distributed proportionally among the two groups **(Table 1)**.

Table 1: Distribution of sample size for household survey

**Primary study population**	**Total number of individuals**	**Percentage (%)**	**Sample size**	**Sample size considering 10% non-response**
Adolescent girls aged 12 to 17 years	60,084	22.3%	88	99
Women aged 18 to 59 years	209,261	77.7%	307	341
Total number of women and adolescent girls aged 12 to 59 years	269,345	100%	395	440

Sampling Techniques: A multistage sampling technique will be employed for selecting camps and study respondents.

Stage I: In the first stage sampling, refugee camps in Ukhiya and Teknaf sub-districts where new makeshifts were established after the August 25, 2017 influx will be selected. Registered refugee camps with old settlements where Rohingya refugees are living since before the influx of August 25, 2017 will be excluded. Eleven camps will be randomly selected from 34 camps; that represents 30% of the total camps in Ukhiya and Teknaf sub-districts at Cox's Bazar. The samples will be equally distributed among the 11 selected camps (40 sample per camp). Our randomly selected camps in the Ukhiya Upazila are Camp-1W, Camp-3, Camp-5, Camp-7, Camp-10, Camp-11 and Camp-17 and in the Teknaf Upazila are Camp- 21, Camp-22 & Camp-26.

Stage II: In the 2nd stage sampling, a complete list of Majhiis (local community leaders of Rohingyas) of the selected 11 camps will be collected from the Camp in-Charge (CiC) office and one Majhii will be randomly selected per camp.

Stage III: Survey data will be collected from 31 women and 9 adolescent girls aged 12-17 years from each camp considering proportionality in these groups.The house of the selected Majhii will be determined as starting point to select the sample households. Selected from both left and right side, the 1st (closest) sample household will be after 10 footsteps from Majhii's house. Accordingly, households in every 10 footsteps will be selected until desired number of

For peer review only - http://bmjopen.bmj.com/site/about/guidelines.xhtml

respondents are interviewed. One woman will be picked from every selected household as a respondent. If more than one woman is found in a household, then one will be randomly selected on the basis of availability and interest for interviewing. In addition, if available, an adolescent (aged 12-17) will be interviewed from the same household. If more than one adolescent girl is found in that household, then similar procedure will be followed.

Data collection methods and tools: A structured quantitative questionnaire (Suppl File. 1) will be prepared following a guideline prepared by UNFPA and Save the Children for humanitarian crisis situation[16] and scholarly literatures.[17] Pretesting will be done in similar camps that are not selected for the study before initiating data collection. Data will be collected through Samsung tablets (Model no. SM-T231) by using SurveyCTO software, an Open Data Kit (ODK) tool widely used for collecting survey data. Using ODK will ensure automatic data storage in database which will be converted to statistical software package Stata 13 (StataCorp, 4905 Lakeway Drive, College Station, Texas, USA) for data cleaning and analyzing. The questionnaire will be translated in Bangla from English including key SRHR terms in Rohingya language. Local dialects will be used in the questions for clarity of our study objective related topics (local language). Local experienced female interviewers, who understand the language of Rohingya community, will be recruited for data collection. Extensive training sessions will be conducted to orient and train them regarding the study objective and tools. The interviewers will be monitored by two researchers and one statistician of BRAC JPGSPH while collecting data. In addition, two local male interviewers will be recruited for building rapport with the community key persons in each camp.

Data analysis: Descriptive analyses will be performed on survey data collected to understand socio demographic characteristics, need for SRH services, health care and service seeking behavior, service utilization patterns and barriers to accessing services, challenges faced on the basis of distance and waiting time at health facilities, their restrictions and reasons for not taking services etc. Statistical analysis will be performed in separate groups for women (18- 59 years) and adolescent girls (12-17 years) to understand their specific SRH needs. Depending on the distribution of variables, frequencies, percentages, mean (standard deviation) and range as summary statistics will be reported. Chi-square test will be performed to measure the association between socio-demographic characteristics and other variables of menstrual health, pregnancy, delivery care, family planning services, menstrual regulation, abortion and sexual transmitted diseases, and feasible challenges of demand side and pattern of health seeking behavior for sexual and reproductive healthcare. Multivariable regression analysis will be conducted afterwards if significant associations are found in bivariate analyses.

Qualitative interviews and group discussions: In order to complement the household survey findings, we will also conduct in-depth interviews (IDIs) with adolescent girls and women. This will help to further understand their perspectives about the SRH services available, the challenges they face in accessing and utilizing those services. IDIs with formal and informal healthcare providers who are working in the selected refugee camps will be done to understand the barriers and challenges in providing SRH services to the Rohingya refugee adolescent girls and women. To understand the perspective of their male counterparts, focus group discussions (FGDs) with Rohingya males will also be conducted. Key informant interviews (KIIs) with the

For peer review only - http://bmjopen.bmj.com/site/about/guidelines.xhtml

key stakeholders from government, INGOs and NNGOs will be conducted to get insights about the existing SRH service delivery system and management challenges. Rohingya community leaders such as Majhees, religious leaders such as Imams and teachers will also be interviewed (KII) to explore their influence on the adolescent girls and women in utilizing SRH services.

Sample size and sampling techniques: For qualitative interviews, three camps will be selected from eleven camps (where household survey and facility assessment will be conducted) depending on geographic location, challenging terrains, remoteness, difficulty in accessibility, availability of infrastructure etc. The sampling strategy, and type and number of respondents for each of the qualitative activities planned is provided in **Table 2**.

Table 2. Sampling frame and characteristics for each qualitative activity

Activity & Focus	Sampling Strategy	Respondent Groups	Estimated Numbers
Activity 1: KIIs	Opportunistic/ emergent sampling<br><br>Snowball sampling	Local and international NGO programme leads, managers, SRH focal points, influential workers, government high officials, programme managers	5
		Influential community members: Majhii, imam, lady imam, Burmese teachers	9
Activity 2: IDIs	Purposive sampling	Rohingya women aged 18-59 years	9
		Rohingya adolescent girls (12-17 years old)	9
	Opportunistic/ emergent sampling	Formal providers such as midwife, health center in-charge, doctors, nurses, community health workers	11
		Informal providers such as traditional birth attendants (TBAs), Burmese doctor, traditional healers,	9
Activity 3: FGDs	Purposive sampling	Rohingya adult males	3 FGDs with maximum 6 participants

Qualitative data collection will be continued till data saturation is achieved.

Data collection methods and tools: Separate guidelines will be developed for IDIs and KIIs with different groups and FGDs with males. All tools will be finalized after pretesting in similar camps that are not selected for this study. Qualitative interviews will be conducted by an experienced group of researchers trained in qualitative interviewing and analysis. However, due to language barrier, interpreters will be recruited from the nearby locality who understand the

For peer review only - http://bmjopen.bmj.com/site/about/guidelines.xhtml

language and dialect of Rohingya community. Training sessions will be conducted to orient and train them on study objective and qualitative tools prior interviewing. A period of rapport building with the community key persons in each camp site will be critical to the success of this research given known difficulties in accessing the Rohingya population, their conservative cultures, suspicion about motives and post-interview repercussions. Interviews with women and adolescent girls will only be conducted by female researchers and interpreters due to conservative nature of the local population and nature of the questions involved. These dynamics must be handled carefully, or else access will be hampered. Networks with influential and key locals will be important in opening doors and initiating discussion. Male researchers including local male interpreters will conduct FGDs, IDIs and KIIs with community males.

Data analysis: An outline of the plan for qualitative data analysis will be prepared in advance of the research which will include defining a priori codes according to study objectives. All interviews will be recorded provided consent has been obtained, along with simultaneous note-taking in case of equipment failure. Data transcription will occur immediately following each interview, followed by translation. Data familiarization will involve reading transcripts repeatedly to surface emerging themes, assess strengths and weaknesses of interview techniques, and identify any missed opportunities for further exploration. Transcripts will be reviewed carefully, and coding will be done following the a priori code-list. A team approach to analysis will be employed to minimize individual biases. Intra-coder and inter-coder reliability will be checked. This approach is applied in all aspects of analysis including coding, with multiple analysts coding the same sections of text to assess inter-coder reliability. Emerging themes and patterns in the data will be tested using data displays that allow more systematic analysis of the qualitative data. Any emerging codes identified during analysis will be added in the code-list after confirmation as a team and will be used for coding all transcripts.

Facility assessment: A facility assessment will be undertaken to get an overview about the supply side barriers in terms of infrastructures, human resources including training needs, provision and utilization of SRH services, and medical supplies for serving Rohingya refugee population. This facility assessment exercise will help assess facility readiness to provide comprehensive SRH services. An estimation of resources required for providing comprehensive SRH services in the camps will also be done with the data from facility assessment and secondary sources.

Sample size and sampling techniques: Five categories of health facilities will be chosen according to WHO Health Facility Register (shared internally by WHO). The categories include primary health centers (PHC), health posts (fixed and plus), labor rooms or SRH only facilities, secondary health facility and community clinics. The first three types of facilities (PHCs, health posts plus and fixed health post, and labor rooms/ SRH only facilities/maternity centers) are camp-specific and situated inside the camp. The two other types of facilities (secondary hospitals and community clinics are situated outside the camps. One facility from each category will be randomly selected for assessment in each camp depending on the availability. In doing so, health facility listing of the selected camps for this study will be carried out from the WHO Health Facility Register. In the camps, if one facility is available in a selected category, then that facility will be assessed. For example: if there is only one PHC in a specific camp, then assessment of that particular facility will be conducted. In some cases, there are more than one facility of a

For peer review only - http://bmjopen.bmj.com/site/about/guidelines.xhtml

single category available inside one camp (such as three PHCs in one camp). In such situation, one facility will be selected for this study using random selection method.

With this procedure the following facilities in **Table 3** will be assessed in the Ukhiya and Teknaf upazila.

Table 3. Types and numbers of facilities for Facility Assessment

**Upazila**	**Camp Name/ Number**	**Categories of health facilities**				
		Health Post	Primary Health Care (PHC) Facility	Labor room or SRH only	Secondary Health Facility	Community Clinic
**Ukhiya**	Camp–1W	1	–	1	4	1
	Camp–3	1	1	1		
	Camp–5	1	1	–		
	Camp–7	1	1	–		
	Camp–10	1	1	–		
	Camp–11	1	1	–		
	Camp–17	1	1	–		
	Kutupalong RC	1	1	–		
**Teknaf**	Camp–21	1	1	–	1	1
	Camp–22	1	1	–		
	Camp–26	1	–	–		
**Sub-total**		**11**	**9**	**2**	**5**	**2**

For peer review only

For secondary health facilities, in both sub-districts, two government secondary facilities (Upazila Health Complex) are serving as the main referral points along with other secondary facilities. Additionally, 2 community clinics are GoB-run primary level facilities under Ministry of Health and Family Welfare. All these health facilities will be selected for assessment. Therefore, we will be conducting facility assessments in total 29 health facilities – 11 health posts (fixed), 9 PHCs, 2 labor rooms and SRH only facilities, 5 secondary health facilities and 2 community clinics altogether (**Table 3**).

<u>Data Collection methods and tools:</u> A structured English questionnaire (Suppl File. 2) will be prepared for facility assessment following WHO Service Availability and Readiness Assessment (SARA) tool.[18] After pretesting and finalizing, two researchers will collect data from different categories of health facilities identified. Data will be collected through Samsung tablets (Model no. SM-T231) by using KOBO software, an ODK tool.

<u>Data analysis:</u> Descriptive analysis will be conducted according to the type of facilities to understand the facility readiness and challenges faced by supply side. The analysis will be performed separately for five different categories proposed to identify gaps at all levels - service provision and availability, service utilization, human resources including their training,

For peer review only - http://bmjopen.bmj.com/site/about/guidelines.xhtml

infrastructure, and supply of equipment and drugs for providing SRH services by the health facilities.

Data Triangulation

This concurrent mixed methods study aims for results point of integration as identified by Schoonenboom & Johnson (2017).[19] Data collected from multiple sources such as household survey, facility assessment, qualitative interviews (IDIs & KIIs) and discussions (FGDs) will be triangulated to understand the overall SRH needs, demands, challenges, barriers to access and service provision to the Rohingya refugee women and adolescent girls aged 12-59 years. After initial descriptive analysis of each qualitative and quantitative component, integration of different components to link and explain different dimensions such as SRH needs, SRH service utilization, barriers to access SRH service etc. will be made. A joint display of qualitative and quantitative findings will also be adopted.

Patient and public involvement

The research questions and outcome measures of this study were chosen based on the priority of comprehensive information need on the Rohingya diaspora in a complex humanitarian condition in discussion with relevant stakeholders including possible comprehensive SRH service implementers and policymakers. However, neither patients nor public were involved in developing this study protocol.

ETHICS AND DISSEMINATION

This study has received ethical approval from Institutional Review Board (IRB) of BRAC James P Grant School of Public Health (2018-017-IR) and poses no more than minimal risk to subjects. Respondents will be asked for written consent prior interviewing. Written assents will be taken from adolescent respondents and written consents will be sought from their guardians. In case of low literacy of respondents, verbal assent and consent will be sought.

Tape recorders will be used for recording the qualitative interviews in order to collect full and intact thoughts. Strong password-protected server or user profile will be created and utilized for quantitative data collection using ODK tools - SurveyCTO for survey and KoBo Toolbox for facility assessment. All forms of data related to this study will be stored in locked storage or controlled-access folders allowing access by authorized persons related to the study i.e. Principal Investigator, other study investigators, and IRB members of BRAC JPGSPH.

Findings from this research will be disseminated at various levels so that evidence generated can be advocated and translated into policy actions for better SRH of Rohingya refugee women and adolescent girls. This study aims to strengthen SRH service provision through incorporating changes recommended from this research findings, for example, on programme implementation, challenges reported by study population and service providers etc. Policy briefs with recommendations specific to programme implementation and related challenges will be developed and disseminated to all higher-level stakeholders. Stakeholder engagement (Ministry of Health and Family Welfare, Government of Bangladesh; Health sector headed by WHO and SRH sub-sector by UNFPA) since inception of the study will be beneficial in influencing local

For peer review only - http://bmjopen.bmj.com/site/about/guidelines.xhtml

policy on SRH service provision. These actors are expected to take lead for service provision changes proposed by the situation analysis and implement necessary amendments in local policy strengthening. Findings will also be presented to relevant local administrators, development partners and NGOs and other relevant parties, academicians and researchers through local and national conferences, dissemination workshops, interactive project report and policy briefs. Additionally, scholarly publications in peer-reviewed journals and presentations in international scientific forums, conferences, and symposiums will be done for international audiences.

EXPECTED CHALLENGES

First and foremost, the challenge expected by researchers of this study is the language barrier as Rohingya people cannot speak or understand Bengali or English languages. Hence, it will be difficult to understand their dialect for the researchers unless trained interpreters are involved. Thus, we will recruit data collectors cum interpreters from the local Bangladeshi community who can speak and understand Rohingya language fluently. They will act as key persons to establish communication between researchers and Rohingya people. However, a list of key terms in Rohingya language and translation into Bangla and English will be prepared with the help of data collectors as a reference for the researchers who will be accompanying the local data collectors to combine their critical thinking with the community dialect. They will also perform spot-checks while collecting data. Another challenge is the sensitive nature of the questions to be asked in this study given the conservative culture of the Rohingya population. Only female data collectors and researchers will interview female respondents and male data collectors and researchers will conduct interviews and FGDs with male respondents. Data collectors will be trained to respect the current humanitarian crises condition in the Rohingya camps, the trauma this community has experienced, their culture, choices and privacy of the interview respondents. Another major challenge can be the entry into local Rohingya community. Harnessing the power relations and dynamics in the Rohingya community is the key to conduct such situation analysis. Thus, the study is designed accordingly to draw on the benefits of engaging gatekeepers to create access in the community for data collection. After entering in each camp, the researchers will communicate with the selected Majhiis, the local Rohingya leaders, and explain him/her the purpose of the study. After leveraging sufficient time to build rapport with the Majhiis, their support will be sought to get access to the households. In terms of working in facilities, accessing the records could be difficult, hence, the higher authority of each facility will be informed beforehand. Another challenge in this study can be political unrest like strike for which data collection may delay. Finally, weather and geographical difficulties is one of the biggest challenges in working at Rohingya camps as located in hilly areas and challenging terrains. In order to avoid any sort of accident, adequate logistics support to the data collectors and study researchers will be ensured.

For peer review only - http://bmjopen.bmj.com/site/about/guidelines.xhtml

Contributors

RA, NF, BA, RH, SBS, PR, AA, AR, MTH, ZQ, SFR contributed in conceptualization or design of the study and VU, LHK, JR, LS reviewed and incorporated their critical inputs. RH, SBS and PR have contributed equally. Also, VU, LHK, JR and LS made equal contributions. RA drafted the initial version with support from NF and BA. MTH, ZQ, SFR reviewed and helped revise critically. RH, SBS, AA, PR, AR, VU, LHK, JR, LS reviewed critically for important intellectual content. RA finally revised the version submitted with inputs from all other co-authors. All authors finally approved the version published. RA, BA, MTH, ZQ, SFR, LHK are in agreement to be accountable for all aspects of the work in ensuring that questions related to the accuracy or integrity of any part of the work are appropriately investigated and resolved.

Competing interests

The authors declare no competing interests.

Funding

Funding is received from World Health Organization (WHO) for this work; Grant Number: 201991701 and Unit Reference - FWC/RHR

Patient consent

Not required

Ethics approval

Institutional Review Board (IRB) of BRAC James P Grant School of Public Health.

For peer review only

REFERENCES

1. Sustainable Development Goals. Goal 3: Ensure healthy lives and promote well-being for all at all ages. United Nations. Available from: https://www.un.org/sustainabledevelopment/health/
2. World Health Organization. Refugee and migrant health. *Reports on situation analysis and practices in addressing the health needs of refugees and migrants.* Available from: http://www.who.int/migrants/publications/situation-analysis-reports/en/
3. Robinson V (Ed.). The international refugee crisis: British and Canadian responses: Springer; 2016. Palgrave Macmillan; 1st ed. 1993, edition: 2016. DOI: 10.1007/978-1-349-12054-3
4. United Nations High Commissioner for Refugees (UNHCR). *Global Trends: Forced Displacement in 2017.* 2018 June 25. Available from: http://www.unhcr.org/statistics/unhcrstats/5b27be547/unhcr-global-trends-2017.html [Accessed on July 30, 2018].
5. World Health Organization. Environmental health in emergencies. Displaced people. https://www.who.int/environmental_health_emergencies/displaced_people/en/ [Accessed on July 31, 2018]
6. World Health Organization. *2015 WHO Humanitarian Response.* Department for Emergency Risk Management and Humanitarian Response (ERM). 2015. Available from: http://www.who.int/hac/who_humanitarian_response_plans2015.pdf [Accessed on July 30, 2018].
7. United Nations Population Fund (UNFPA). *Protecting Women in Emergency Situations.* Available from: https://www.unfpa.org/resources/protecting-women-emergency-situations [Accessed on August 1, 2018].
8. Haque MM. Rohingya Ethnic Muslim Minority and the 1982 Citizenship Law in Burma. *Journal of Muslim Minority Affairs* 2017;37(4):454-69.
9. Khatun F. *Implications of the Rohingya Crisis for Bangladesh.* Centre for Policy Dialogue (CPD): Dhaka, Bangladesh, 2017 November 11.
10. International Organization for Migration (IOM). *Needs and Population Monitoring (NPM). Site Assessment: Round 10; 2018.* May 20, 2018.
11. *Rohingya Crisis in Cox's Bazar, Bangladesh Health Sector Bulletin#4.* Health Sector Coordination team April 12, 2018. Available from: http://www.searo.who.int/bangladesh/healthsectorcxbbanbulletinno4.pdf?ua=1 [Accessed on July 30, 2018].
12. Doedens W, Burns K. *Challenges to reproductive health in emergencies.* Health in Emergencies. Department of Emergency and Humanitarian Action. World Health Organization (WHO). Issue 10, June 2001;P 1-7.
13. Reliefweb. *Bangladesh Refugee Emergency Population Factsheet* (as of 30 June, 2018). Available from: https://reliefweb.int/sites/reliefweb.int/files/resources/64651.pdf
14. United Nations Population Fund Bangladesh (UNFPA). *UNFPA Rohingya Humanitarian Response. Monthly Situation Report.* May 2018. Available from: https://bangladesh.unfpa.org/sites/default/files/pub-pdf/UNFPA_SitRep_External__17%20May%202018%20Final.pdf
15. Cochran WG. Sampling techniques. John Wiley & Sons. 2007;Pg. 24. Available from:

For peer review only - http://bmjopen.bmj.com/site/about/guidelines.xhtml

http://krishikosh.egranth.ac.in/bitstream/1/2034365/1/CIFE-100.pdf

16. UNFPA and Save the Children. *Adolescent Sexual and Toolkit for Reproductive Health Humanitarian Settings: A Companion to the Inter-Agency Field Manual on Reproductive Health in Humanitarian Settings*. 2009. 99 p.
17. Pyone T, Dickinson F, Kerr R, et al. Data collection tools for maternal and child health in humanitarian emergencies: a systematic review. *Bull World Health Organ* 2015;93(9):648–658A. Available from: http://www.who.int/entity/bulletin/volumes/93/9/14-148429.pdf
18. World Health Organization (WHO). *Service Availability and Readiness Assessment (SARA). An annual monitoring system for service delivery. Reference manual. Version 2.1.* September 2013. Available from: https://www.who.int/healthinfo/systems/SARA_Reference_Manual_Full.pdf
19. Schoonenboom J & Johnson R. B. How to construct a mixed methods research design. *Kolner Z Soz Sozpsychol* 2017;69(Suppl 2):107-131. Available from: https://link.springer.com/content/pdf/10.1007%2Fs11577-017-0454-1.pdf

For peer review only

For peer review only - http://bmjopen.bmj.com/site/about/guidelines.xhtml

Figure 1: Sampling techniques to conduct situation analysis

Supplementary File 1: Household Survey Questionnaire

Supplementary File 2: Facility Assessment Questionnaire

For peer review only

For peer review only - http://bmjopen.bmj.com/site/about/guidelines.xhtml

Figure 1: Sampling techniques to conduct situation analysis

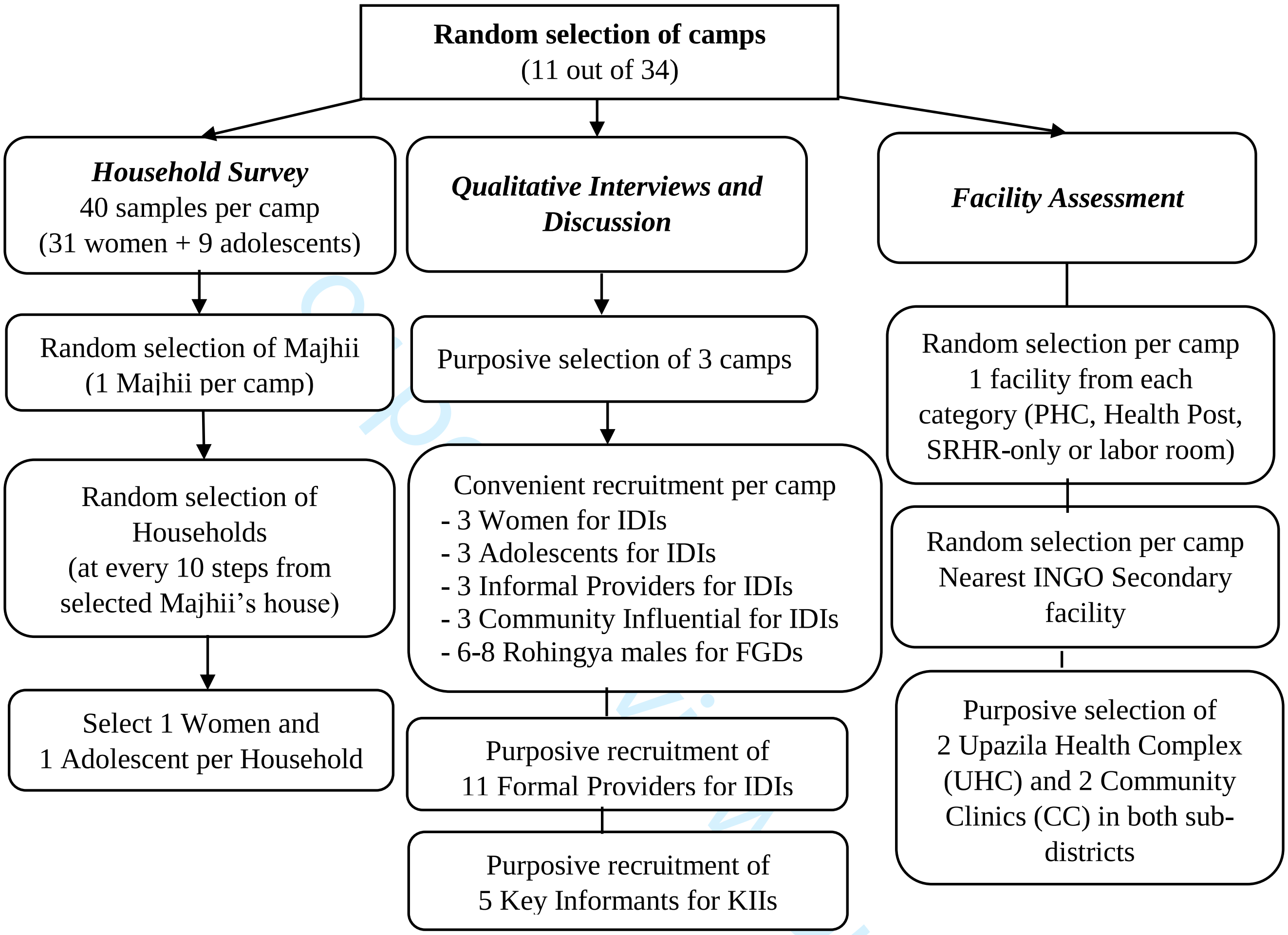

Survey Questionnaire
Situation Analysis for Delivering Integrated Comprehensive SRHR Services for Rohingya Refugees in Cox's Bazar, Bangladesh

Section 1: General Information about the respondent

Participant ID:<br>(ID of camp, ID of block, ID of Majhee, respondent number start with 001)	
Camp name/number	
Block number	
Name of Majhee (ID of Majhee)	
Name of household head	
Household number	
Interview start time	
Interview end time	

Section 2: Socio-demographic characteristics

For peer review only

No.	Questions and instructions	Coding categories	Skip
2.1	When did you first arrive at Cox's Bazar/Bangladesh?	Months |__|__| Years |__|__|	
2.2	When did you first arrive at this particular camp?	Months |__|__| Years |__|__|	
2.3	How old are you?<br>(*Instruction: If she could not tell her age, then <u>if married</u> - ask how many years she has been married, approximate age at marriage, approximate age during 1st menstruation, age of 1st child/approximate age at 1st pregnancy, after how many years of marriage she got pregnant for the 1st time. <u>If unmarried</u>- ask approximate age during 1st menstruation, how many years having menstruation, years of schooling, age at enrollment in school, study gap in years*)	Years |__|__|	
2.4	What is your religion?	Muslim…………………………….....1<br>Buddhist ……………………………2<br>Hindu ……………………………….3<br>Christian ……………………………4<br>Others (Please specify ………… 96<br>Did not answer ………………….. 99	
2.5	What is your marital status?<br>*(If husband is currently living with her or not, or the husband got married to another place and live separately- these things need to be known by asking)*	Married...……..……………………..1<br>Unmarried …………………………2<br>Widow ………..……………………3<br>Married; husband stays in Myanmar..4<br>Married; husband stays Abroad……..5<br>Married; husband stays at different place (still in touch)…………………6	

ID of the Participant: |___|___|___|___|___|___|___|___|

For peer review only - http://bmjopen.bmj.com/site/about/guidelines.xhtml

Survey Questionnaire
Situation Analysis for Delivering Integrated Comprehensive SRHR Services for Rohingya Refugees in Cox's Bazar, Bangladesh

No.	Questions and instructions	Coding categories			Skip
		Married; husband is not in touch (No communication in last 1 month)…….7<br>Separated/ Divorced…………………8<br>Did not answer ………………….. 99			
2.6	Who are you currently living with?<br>*(If living with husband and parents/in-laws/relatives, then code as husband. kOtherwise code accordingly)*<br>*(Multiple options could be the answer)*	Alone ………………………………1<br>Husband ……………………………2<br>Children (Son/Daughter)……………3<br>Brother/Sister ……………………… 4<br>Parents/in-laws …………………….. 5<br>Relatives …………………………… 6<br>Did not answer ………………….. 99			1 → 2.10
2.7	How many people are currently living in your house/tent??	Number of people |__|__|			
2.8	Among those members, how many are aged less than 18 years?	Number of people |__|__|			
2.9	Who is the sole decision maker in your family? [Probe: Makes the important decisions of the family]<br>- Marriage of children/education<br>- Spending money<br>- Any important decision in family		Yes	No	
		Myself	1	0	
		Husband	1	0	
		Father	1	0	
		Mother	1	0	
		Brother	1	0	
		Other Relative	1	0	
		Majhi	1	0	
		Others (Specify) ………………..………… 96			
		Did not answer……………………………… 99			
2.10	Can you read? For example, a newspaper, book etc.?	Yes ……………………………….1<br>No …………………………………2<br>Did not answer ………………….. 99			2 → 2.13<br>99 → 2.13
2.11	Have you ever-attended school?	Yes ……………………………….1<br>No …………………………………2<br>Did not answer ………………….. 99			2 → 2.13<br>99 → 2.13
2.12	What is the highest level of education you completed? [Check with Ques 2.10 to be consistent, and correct Ques 2.10 if necessary]<br>- Have you completed the examination for the class you have studied highest?	Primary ……………………………1<br>Secondary …………………………2<br>University …………………………3<br>Post Graduate ……………………..4<br>Did not answer ………………….. 99			

ID of the Participant: |___|___|___|___|___|___|___|___|

For peer review only - http://bmjopen.bmj.com/site/about/guidelines.xhtml

Survey Questionnaire
Situation Analysis for Delivering Integrated Comprehensive SRHR Services for Rohingya Refugees in Cox's Bazar, Bangladesh

No.	Questions and instructions	Coding categories	Yes	No	Skip
2.13	What type of work you are engaged for earning money?<br>Please probe with each option mentioned (Multiple Response)		Yes	No	
		Housekeeping (at other's home/hotel)	1	0	
		Works at Field	1	0	
		Catches Fish	1	0	
		Fuel-wood collection	1	0	
		Construction site labor	1	0	
		Dily Wage Labor (other than 1, 2 and 3)	1	0	
		Own small business (e.g. grocery, selling vegetables, etc.)	1	0	
		Monthly-wage labor (e.g. community health worker, service at health center/WFC/NGO office, etc.)	1	0	
		Fishing	1	0	
		Currently not working	1	0	
		Others (Specify) ………………….………………. 96			
		Did not answer………………..…………….…... 99			
2.14	What is the main source of your family income?<br>[Multiple responses possible]		Yes	No	
		Respondent's income	1	0	
		Relief	1	0	
		Fuel-wood collection	1	0	
		Daily wage labor	1	0	
		Own small business (e.g. grocery, selling vegetables, etc.)	1	0	
		Monthly-wage labor (e.g., teacher, other service, etc.)	1	0	
		Others (Specify) ………………….………………. 96			
		Did not answer………………..…...……………. 99			
2.15	What is your family's monthly expenditure in last month?<br>*Probe: (ask for approximate amount for*<br>- *Food*<br>- *Clothes*<br>- *Education*<br>- *Other expenses*)	Taka |__|__|__|__|__|			

ID of the Participant: |___|___|___|___|___|___|___|___|

For peer review only - http://bmjopen.bmj.com/site/about/guidelines.xhtml

Survey Questionnaire
Situation Analysis for Delivering Integrated Comprehensive SRHR Services for Rohingya Refugees in Cox's Bazar, Bangladesh

Type of Participant:

(Surveyor will fill this up according to the age of the respondent mentioned in the question 2.3)

- Women
- Adolescent

Based on the category next section of questions will be appeared.

Section 3: Menstrual health (only for adolescent girls aged 14-17)

No.	Questions and instructions	Coding categories			Skip
3.1	Has your menstruation started?	Yes ……………………………………1			
		No ……………………………….…2 →			5.3
		Did not answer…………………… 99 →			5.1
3.2	How many months/years back, did you have your first menstruation?	Number of Months |__|__|			
3.3	What do you usually use during menstruation? (Multiple Response)		Yes	No	
		Cloth	1	0	
		Cotton	1	0	
		Sanitary Napkin	1	0	
		Toilet Paper	1	0	
		Others (Specify) ……………..…………… 96			
3.4	Do you have any of the symptoms when you have periods? MULTIPLE RESPONSE		Yes	No	
		Abdominal pain	1	0	
		Lower back pain	1	0	
		Pain in upper leg and thighs	1	0	
		Nausea	1	0	
		Tiredness	1	0	
		Body-aches	1	0	
		Dizziness	1	0	
		Headaches	1	0	
		Joint pains	1	0	
		Tender breasts	1	0	
		No symptoms	1	0 →	3.8
		Others (Specify)……………….…………… 96			
		Did not answer…………………………… 99			

ID of the Participant: |___|___|___|___|___|___|___|___|

For peer review only - http://bmjopen.bmj.com/site/about/guidelines.xhtml

Survey Questionnaire
Situation Analysis for Delivering Integrated Comprehensive SRHR Services for Rohingya Refugees in Cox's Bazar, Bangladesh

No.	Questions and instructions	Coding categories	Yes	No	Skip
3.5	What do you do when you have any of those symptoms? MULTIPLE RESPONSE		Yes	No	
		Take rest	1	0	
		Drink warm water	1	0	
		Take hot shower/ hot biting	1	0	
		Take medicine from local drug-store	1	0	
		No measures taken	1	0	
		Others (Specify)…………………….……………. 96			
		Did not answer……………………………… 99			
3.6	Have you ever consulted with anyone about the symptoms you have during menstruation?		Yes	No	
		Didn't consult with anyone	1	0	3.8
		Mother/sister/family member	1	0	3.8
		Husband	1	0	3.8
		Relative	1	0	3.8
		Friends	1	0	3.8
		Neighbor	1	0	3.8
		Health Worker visited home	1	0	3.8
		Doctor/Nurse/Paramedic	1	0	
		Local Traditional healer	1	0	3.8
		Others (Specify)…………….……………….. 96			3.8
		Did not answer……………………………… 99			3.8
3.7	Where did you go to consult with doctor/nurse/paramedic? (Instruction: If the respondent says any name, probe if that is healthcare facility)	Healthcare Facility (Specify) ……...1<br>Others (Specify)…………………. 96<br>Didn't answer …………………….99			
3.8	Do you have restriction to any certain behavior during your period? (Multiple Response)		Yes	No	
		Restricted mobility outside home	1	0	
		Certain places are restricted to go (e.g. kitchen, graveyard, fetching water/ponds, etc.)	1	0	
		Certain foods are restricted (e.g. certain type of fish, meat, sour etc.)	1	0	
		Separate sleeping area	1	0	
		Not allowed to sweep the house	1	0	
		Not allowed to keep the hair untied	1	0	

ID of the Participant: |___|___|___|___|___|___|___|___|

For peer review only - http://bmjopen.bmj.com/site/about/guidelines.xhtml

Survey Questionnaire
Situation Analysis for Delivering Integrated Comprehensive SRHR Services for Rohingya Refugees in Cox's Bazar, Bangladesh

No.	Questions and instructions	Coding categories			Skip
		No restrictions	1	0	
		Others (Specify) ……………………………96			
		Didn't answer …………………………… 99			

Section 4: Pregnancy and Delivery care

No.	Questions and instructions	Coding categories			Skip
4.1	Have you ever given birth?	Yes ………………………………1<br>No ………………………………2<br>Didn't answer ……………………99			Next section
4.2	How many of your son live with you at your house currently?	Number of sons |__|__|			
4.3	How many of your daughter live with you at your house currently?	Number of daughters |__|__|			
4.4	How many son live separated from you?	Sons live elsewhere |__|__|			
4.5	How many daughters live separated from you?	Daughters live elsewhere |__|__|			
4.6	How many of your boy child was born alive but died?<br>*[If none, put 00 in two boxes]*	Boys dead |__|__|			
4.7	How many of your girl child was born alive but died?<br>*[If none, put 00 in two boxes]*	Girls dead |__|__|			
4.8	How many miscarriage or Menstrual Regulation you had?<br>*[If none, put 00 in two boxes]*	Number of pregnancies terminated |__|			
4.9	How many still births did you had?	Number of dead child/children |__|			
4.10	Total number of pregnancies?	|__|__|			
	**Now I would like to ask you about your pregnancy history after coming here (Bangladesh/Cox's Bazar)**				
4.11	Are you pregnant now?	Yes ………………………………...1<br>No ………………………………2<br>Did not answer……………………...99			 4.23<br>4.23
4.12	For How many months in your pregnancy are you at?	Number of months |__|__|			
4.13	Are you following up with anyone for antenatal care for this pregnancy?	Yes ………………………………...1<br>No ………………………………2<br>Didn't answer ……………………99			 4.18<br>4.19
4.14	Who are you following up with?<br>Probe to identify each type<br>[Multiple response possible]		Yes	No	
		Health worker visited home	1	0	
		Local TBA at home	1	0	
		Doctor/Nurse/Midwife/Paramedic at a health facility/clinic	1	0	

ID of the Participant: |___|___|___|___|___|___|___|___|

For peer review only - http://bmjopen.bmj.com/site/about/guidelines.xhtml

Survey Questionnaire
Situation Analysis for Delivering Integrated Comprehensive SRHR Services for Rohingya Refugees in Cox's Bazar, Bangladesh

No.	Questions and instructions	Coding categories			Skip
		Burmese /Private Doctor at a health facility/clinic	1	0	
		Drug-shop/Pharmacy	1	0	
		Others Specify …………………96			
		Didn't answer …………………99			
4.15	Where did you receive antenatal care for this pregnancy?<br>(Instruction: If the respondent says any name, probe if that is healthcare facility)	Home ………………………………1<br>Healthcare Facility (Specify) ……...2<br>Others (Specify)………………… 96<br>Didn't answer ……………………99			
4.16	How many antenatal care visits did you receive during this pregnancy?	Never …………………………… 1 →<br>Once …………………………… 2<br>Twice …………………………… 3<br>Four Times ……………………… 4<br>Others (Specify)…………...…. 96<br>Don't know . . . . ………….98<br>Didn't answer ……………..... 99			4.18
4.17	As part of your antenatal care during this pregnancy, were any of the following done at least once?<br>• Was your weight measured?<br>• Was your blood pressure measured?<br>• Did you have a urine test?<br>• Did you have a blood test?<br>• Did you have an ultrasonography?<br>• Were you counselled about danger signs?<br>• Was your fundus height checked? (Did they use any machine to check your abdomen?)		Yes	No	
		Weight	1	0	
		Height	1	0	
		Blood Pressure	1	0	
		Urine	1	0	
		Bloodtest	1	0	
		Ultrasound	1	0	4.19
		Advice Danger Sign	1	0	
		Abdominal Test	1	0	
		Titanus (TT)	1	0	
		Others	1	0	
		Don't Know ……………………98			
		Didn't Answer …………………99			
4.18	(If you didn't receive antenatal care), What was the reason for not receiving antenatal care during this pregnancy?		Yes	No	
		Did not feel the necessity	1	0	
		Did not know where/who to go	1	0	
		Did not know such care	1	0	
		Health center is far/Access to facility was/is difficult	1	0	
		Services were/are not available here	1	0	
		Did not like services provided in the facilities	1	0	
		Family/Husband did not allow	1	0	
		Did not have money	1	0	
		Nobody to accompany	1	0	
		Previous experience is not good	1	0	
		Concerned about being treated by a male doctor	1	0	
		Afraid to go health care center	1	0	

ID of the Participant: |___|___|___|___|___|___|___|___|

For peer review only - http://bmjopen.bmj.com/site/about/guidelines.xhtml

Survey Questionnaire
Situation Analysis for Delivering Integrated Comprehensive SRHR Services for Rohingya Refugees in Cox's Bazar, Bangladesh

No.	Questions and instructions	Coding categories			Skip
		Hadn't time	1	0	
		Neighbor forbade to go	1	0	
		Health care center was far away from home	1	0	
		Others (Specify) …………………96			
		Didn't Answer …………………..99			
4.19	During this pregnancy, do /did you have any pregnancy related problems or complications? (after arriving in this camp) MULTIPLE RESPONSE		Yes	No	
		Bleeding	1	0	
		Fever	1	0	
		Frequent Vomiting	1	0	
		Headache/ Blurred Vision	1	0	
		Edema	1	0	
		Convulsion	1	0	
		Abdominal Pain	1	0	
		Less/no movement of the Fetus	1	0	
		No Problem	1	0	→4.23
		Others (Specify) ……………………..96			
		Didn't Answer ……………………….99			
4.20	Did you seek help for the problem(s) or complication(s)?	Yes ………………………………..1<br>No …………………………………..2<br>Didn't answer ……………… 99			 →4.22<br>→4.23
4.21	Where did you seek help?<br>(Instruction: If the respondent says any name, probe if that is healthcare facility)	Home ……………………………….1<br>Healthcare Facility (Specify) ……...2<br>Others (Specify)………………….. 96<br>Didn't answer ……………………..99			4.23
4.22	(If you didn't seek service), What was the reason for not seeking help / service for the complication / problem you had during you're this pregnancy?<br><br>(Multiple Response)		Yes	No	
		Did not feel the necessity	1	0	
		Did not know where/who to go	1	0	
		Did not know such care	1	0	
		Health center is far/Access to facility was/is difficult	1	0	
		Services were/are not available here	1	0	
		Did not like services provided in the facilities	1	0	
		Family/Husband did not allow	1	0	
		Did not have money	1	0	
		Nobody to accompany	1	0	
		Previous experience is not good	1	0	
		Concerned about being treated by a male doctor	1	0	
		Afraid to go health care center	1	0	
		Hadn't time	1	0	

ID of the Participant: |___|___|___|___|___|___|___|___|

For peer review only - http://bmjopen.bmj.com/site/about/guidelines.xhtml

Survey Questionnaire
Situation Analysis for Delivering Integrated Comprehensive SRHR Services for Rohingya Refugees in Cox's Bazar, Bangladesh

No.	Questions and instructions	Coding categories	Skip
		Neighbor forbade to go 1 0<br>Health care center was far away from home 1 0<br>Others (Specify) ……………………..96<br>Didn't Answer ……………………….99	
4.23	Were you pregnant after coming to this camp?<br><br>Instruction: The pregnancy before current pregnancy	Yes ………………………………...1<br>No …………………………………2 →	4.34
4.24	Did you see anyone for antenatal care for last pregnancy?	Yes ………………………………...1<br>No …………………………………2<br>Didn't answer ……………………..99	4.29
4.25	Whom did you see?<br>Probe to identify each type<br>[Multiple response possible]	(Yes / No)<br>Health worker visited home 1 0<br>Local TBA at home 1 0<br>Doctor/Nurse/Midwife/Paramedic at a health facility/clinic 1 0<br>Burmese /Private Doctor at a health facility/clinic 1 0<br>Drug-shop/Pharmacy 1 0<br>Others Specify …………………96<br>Didn't answer …………………99	
4.26	Where did you receive antenatal care for last pregnancy?<br>(Instruction: If the respondent says any name, probe if that is healthcare facility)	Home ………………………………1<br>Healthcare Facility (Specify) ……...2<br>Others (Specify)………………… 96<br>Didn't answer ……………………99	
4.27	How many times did you receive antenatal care during last pregnancy?	Never …………………………… 1 →<br>Once …………………………… 2<br>Twice …………………………… 3<br>Four Times ……………………… 4<br>Others (Specify)…………….... 96<br>Don't know . . . . ………….98<br>Didn't answer ………………..... 99	4.29
4.28	As part of your antenatal care during last pregnancy, were any of the following done at least once?<br>• Was your weight measured?<br>• Was your blood pressure measured?<br>• Did you have a urine test?<br>• Did you have a blood test?<br>• Did you have an ultrasonography?<br>• Were you counselled about danger signs?<br>Was your fundus height checked? (Did they use any machine to check your abdomen?)	(Yes / No)<br>Weight 1 0<br>Height 1 0<br>Blood Pressure 1 0<br>Urine 1 0<br>Bloodtest 1 0<br>Ultrasound 1 0<br>Advice Danger Sign 1 0<br>Abdominal Test 1 0<br>Titanus (TT) 1 0<br>Others 1 0<br>Don't Know ……………………..98<br>Didn't Answer …………………..99	4.30

ID of the Participant: |___|___|___|___|___|___|___|___|

For peer review only - http://bmjopen.bmj.com/site/about/guidelines.xhtml

Survey Questionnaire
Situation Analysis for Delivering Integrated Comprehensive SRHR Services for Rohingya Refugees in Cox's Bazar, Bangladesh

No.	Questions and instructions	Coding categories	Yes	No	Skip
4.29	(If you didn't receive antenatal care), What was the reason for not receiving antenatal care during your last pregnancy?		Yes	No	
		Did not feel the necessity	1	0	
		Did not know where/who to go	1	0	
		Did not know such care	1	0	
		Health center is far/Access to facility was/is difficult	1	0	
		Services were/are not available here	1	0	
		Did not like services provided in the facilities	1	0	
		Family/Husband did not allow	1	0	
		Did not have money	1	0	
		Nobody to accompany	1	0	
		Previous experience is not good	1	0	
		Concerned about being treated by a male doctor	1	0	
		Afraid to go health care center	1	0	
		Hadn't time	1	0	
		Neighbor forbade to go	1	0	
		Health care center was far away from home	1	0	
		Others (Specify) …………………96			
		Didn't Answer …………………..99			
4.30	During last pregnancy, what type of problems or complications you had?<br>MULTIPLE RESPONSE		Yes	No	
		Bleeding	1	0	
		Fever	1	0	
		Frequent Vomiting	1	0	
		Headache/ Blurred Vision	1	0	
		Edema	1	0	
		Convulsion	1	0	
		Abdominal Pain	1	0	
		Less/no movement of the Fetus	1	0	
		No Problem	1	0	→ 4.34
		Others (Specify) ……………………..96			
		Didn't Answer ……………………….99			
4.31	Did you seek help for the problem(s) or complication(s)?	Yes ……………………………….1<br>No …………………………………2<br>Didn't answer ……………… 99			No/Didn't answer → 4.33
4.32	Where did you seek help?<br>(Instruction: If the respondent says any name, probe if that is healthcare facility)	Home ………………………………1<br>Healthcare Facility (Specify) ……...2<br>Others (Specify)…………………. 96<br>Didn't answer …………………….99			
4.33	(If you didn't seek service), What was the reason for not seeking help / service for the complication / problem you had during your last pregnancy?<br><br>(Multiple Response)		Yes	No	
		Did not feel the necessity	1	0	
		Did not know where/who to go	1	0	
		Did not know such care	1	0	

ID of the Participant: |___|___|___|___|___|___|___|___|

For peer review only - http://bmjopen.bmj.com/site/about/guidelines.xhtml

Survey Questionnaire

Situation Analysis for Delivering Integrated Comprehensive SRHR Services for Rohingya Refugees in Cox's Bazar, Bangladesh

No.	Questions and instructions	Coding categories			Skip
		Health center is far/Access to facility was/is difficult	1	0	
		Services were/are not available here	1	0	
		Did not like services provided in the facilities	1	0	
		Family/Husband did not allow	1	0	
		Did not have money	1	0	
		Nobody to accompany	1	0	
		Previous experience is not good	1	0	
		Concerned about being treated by a male doctor	1	0	
		Afraid to go health care center	1	0	
		Hadn't time	1	0	
		Neighbor forbade to go	1	0	
		Health care center was far away from home	1	0	
		Others (Specify) ……………………96			
		Didn't Answer ……………………99			
4.34	Did you give birth to a child after coming here?	Yes ………………………………1<br>No …………………………………2<br>Didn't answer ……………………99			No → Next section
4.35	When did you delivered a child last time?<br>(Less than 1 month and 15 days will be 1 month)	Months ago |__|__|			
4.36	What was the mode of delivery?	Normal vaginal ……………………1<br>Caesarian …………………………..2			
4.37	Is the baby alive?	Yes ………………………………1<br>No …………………………………2<br>Didn't answer ……………………99			
4.38	Where did you give birth?<br>(Instruction: If the respondent says any name, probe if that is healthcare facility)	Home ………………………………1<br>Healthcare Facility (Specify) ……...2<br>Others (Specify)………………… 96<br>Didn't answer ……………………99			2 → 4.41
4.39	Who helped you during your child birth?<br><br>(Multiple Question)		Yes	No	
		Family member/relative/neighbor	1	0	
		Health worker who visited the home	1	0	
		Local traditional healer	1	0	
		Local drug store / Pharmacy	1	0	

ID of the Participant: \|___\|___\|___\|___\|___\|___\|___\|___\|

For peer review only

For peer review only - http://bmjopen.bmj.com/site/about/guidelines.xhtml

Survey Questionnaire
Situation Analysis for Delivering Integrated Comprehensive SRHR Services for Rohingya Refugees in Cox's Bazar, Bangladesh

No.	Questions and instructions	Coding categories			Skip
		Local TBA	1	0	
		Others (Specify)…………………. .96			
		Didn't answer …………………….99			
4.40	**(If you had home delivery)**, What was the reason for not going to any health facility for delivering your child?<br>SKIP IF DELIVERED AT ANY HEALTH FACILITY		Yes	No	
		Did not feel the necessity	1	0	
		Did not know where/who to go	1	0	
		Did not know such care	1	0	
		Health center is far/Access to facility was/is difficult	1	0	
		Services were/are not available here	1	0	
		Did not like services provided in the facilities	1	0	
		Family/Husband did not allow	1	0	
		Did not have money	1	0	
		Nobody to accompany	1	0	
		Previous experience is not good	1	0	
		Concerned about being treated by a male doctor	1	0	
		Afraid to go health care center	1	0	
		Hadn't time	1	0	
		Neighbor forbade to go	1	0	
		Health care center was far away from home	1	0	
		Others (Specify) ……………………96			
		Didn't Answer ……………………..99			
4.41	During last child birth, did you have any complication?	Yes …………………………………… 1<br>No …………………………………… 2<br>Didn't answer ……………………….. 99			2, 99 → 4.45
4.42	What type of problem(s) or complication(s) did you have?<br>MULTIPLE RESPONSE		Yes	No	
		Heavy bleeding	1	0	
		Prolong labor	1	0	
		Obstructed labor	1	0	

ID of the Participant: |___|___|___|___|___|___|___|___|

For peer review only - http://bmjopen.bmj.com/site/about/guidelines.xhtml

Survey Questionnaire
Situation Analysis for Delivering Integrated Comprehensive SRHR Services for Rohingya Refugees in Cox's Bazar, Bangladesh

No.	Questions and instructions	Coding categories			Skip
		Fever	1	0	
		Edema	1	0	
		Convulsion	1	0	
		Retained Placenta	1	0	
		Others (Specify)…………………. 96			
		Do not know…………………….. 98			
		Didn't answer ……………………99			
4.43	Where did you seek help for the problem(s) or complication(s)?<br>(Instruction: If the respondent says any name, probe if that is healthcare facility)	Did not seek any help ……………. 1<br>Home ………………………………2<br>Healthcare Facility (Specify) ……...3<br>Others (Specify)…………………. 96<br>Didn't answer ……………………99			4.45 (Home, Healthcare Facility, Others, Didn't answer)
4.44	(If you didn't seek service), What was the reason for not seeking help / service for the complication / problem you had during delivery?<br><br>MULTIPLE RESPONSE		Yes	No	
		Did not feel the necessity	1	0	
		Did not know where/who to go	1	0	
		Did not know such care	1	0	
		Health center is far/Access to facility was/is difficult	1	0	
		Services were/are not available here	1	0	
		Did not like services provided in the facilities	1	0	
		Family/Husband did not allow	1	0	
		Did not have money	1	0	
		Nobody to accompany	1	0	
		Previous experience is not good	1	0	
		Concerned about being treated by a male doctor	1	0	
		Afraid to go health care center	1	0	
		Hadn't time	1	0	
		Neighbor forbade to go	1	0	
		Health care center was far away from home	1	0	
		Others (Specify) ……………………96			
		Didn't Answer ………………………99			

ID of the Participant: |___|___|___|___|___|___|___|___|

For peer review only

For peer review only - http://bmjopen.bmj.com/site/about/guidelines.xhtml

Survey Questionnaire
Situation Analysis for Delivering Integrated Comprehensive SRHR Services for Rohingya Refugees in Cox's Bazar, Bangladesh

No.	Questions and instructions	Coding categories			Skip
4.45	Did you see anyone for postnatal care after delivery?	Yes ………………………………1<br>No ………………………………2<br>Didn't answer ……………………99			No, Didn't answer → 4.49 Next section
4.46	Whom did you see?		Yes	No	
		Health worker visited home	1	0	
		Local TBA	1	0	
		Doctor/Nurse/ Midwife/paramedic	1	0	
		Burma Doctor/Privet doctor	1	0	
		Drug Store	1	0	
		Others (Specify)…………………… 96			
		Didn't answer ……………………..99			
4.47	Where did you receive postnatal care for this /last pregnancy?<br>(Instruction: If the respondent says any name, probe if that is healthcare facility)	Home ………………………………1<br>Healthcare Facility (Specify) ……...2<br>Others (Specify)………………… 96<br>Didn't answer ……………………99			
4.48	How many times did you receive postnatal care after delivery?	Never………………………………1<br>Once…………………………......2<br>Twice………………………………3<br>Thrice…………………….…...4<br>Four Time………………………5<br>Others…………………………96<br>Don't know …………………98<br>Didn't answer…………………99			Once to Didn't answer → Next Section
4.49	(If you didn't receive postnatal care), What was the reason for not receiving postnatal care after delivery?<br><br>MULTIPLE RESPONSE		Yes	No	
		Did not feel the necessity	1	0	
		Did not know where/who to go	1	0	
		Did not know such care	1	0	
		Health center is far/Access to facility was/is difficult	1	0	
		Services were/are not available here	1	0	

ID of the Participant: |__|__|__|__|__|__|__|__|

For peer review only - http://bmjopen.bmj.com/site/about/guidelines.xhtml

Survey Questionnaire
Situation Analysis for Delivering Integrated Comprehensive SRHR Services for Rohingya Refugees in Cox's Bazar, Bangladesh

No.	Questions and instructions	Coding categories			Skip
		Did not like services provided in the facilities	1	0	
		Family/Husband did not allow	1	0	
		Did not have money	1	0	
		Nobody to accompany	1	0	
		Previous experience is not good	1	0	
		Concerned about being treated by a male doctor	1	0	
		Afraid to go health care center	1	0	
		Hadn't time	1	0	
		Neighbor forbade to go	1	0	
		Health care center was far away from home	1	0	
		Others (Specify) ……………………96			
		Didn't Answer ………………………99			

Section 5: Family Planning services

No.	Questions and instructions	Coding categories			Skip
5.1	Have you or your husband ever used anything to delay or prevent you from getting pregnant?	Yes ………………………………1			
		No …………………………………2			→ 5.3
		Never had physical relation ………3			→ 5.3
5.2	What is/are the method(s) have you ever used?<br><br>CIRCLE ALL MENTIONED (MULTIPLE RESPONSE)		Yes	No	
		Pill	1	0	
		Injection	1	0	
		Condom	1	0	
		IUD	1	0	
		Norplant/Implant	1	0	
		Male sterilization	1	0	
		Female sterilization	1	0	
		Emergency Contraception Pill	1	0	
		Safe period	1	0	
		Withdrawal	1	0	
		Others (Specify)………………… 96			
		Didn't answer ……………………99			

ID of the Participant: |___|___|___|___|___|___|___|___|

For peer review only - http://bmjopen.bmj.com/site/about/guidelines.xhtml

Survey Questionnaire
Situation Analysis for Delivering Integrated Comprehensive SRHR Services for Rohingya Refugees in Cox's Bazar, Bangladesh

No.	Questions and instructions	Coding categories	Yes	No	Skip
5.3	What is your preferred method?		Yes	No	
		Pill	1	0	
		Injection	1	0	
		Condom	1	0	
		IUD	1	0	
		Norplant/Implant	1	0	
		Male sterilization	1	0	
		Female sterilization	1	0	
		Emergency Contraception Pill	1	0	
		Safe period	1	0	
		Withdrawal	1	0	
		Others (Specify)………………… 96			
		Didn't answer ……………………99			
5.4	Why do you prefer this method?	Easy to use ……………………… 1<br>Give me long time protection ……. 2<br>Don't need husband's permission .. 3<br>Adjust with my body …………….. 4<br>Using this since long …………….. 5<br>Others (Specify)………………… 96<br>Didn't answer ……………………99			
5.5	What is/are the method(s) have you heard about?<br><br>(MULTIPLE RESPONSE)		Yes	No	
		Pill	1	0	
		Injection	1	0	
		Condom	1	0	
		IUD	1	0	
		Norplant/Implant	1	0	
		Male sterilization	1	0	
		Female sterilization	1	0	
		Emergency Contraception Pill	1	0	
		Safe period	1	0	
		Withdrawal	1	0	
		Didn't hear anything	1	0	→ Next section
		Others (Specify) ………………… 96			
		Didn't answer……………………99			
5.6	From where/whom this method can be obtained?<br>(Instruction: If the respondent says any name, probe if that is healthcare facility)	Home ……………………………1<br>Healthcare Facility (Specify) ……...2<br>Others (Specify)………………… 96<br>Didn't answer ……………………99			

ID of the Participant: |___|___|___|___|___|___|___|___|

For peer review only - http://bmjopen.bmj.com/site/about/guidelines.xhtml

Survey Questionnaire
Situation Analysis for Delivering Integrated Comprehensive SRHR Services for Rohingya Refugees in Cox's Bazar, Bangladesh

No.	Questions and instructions	Coding categories	Yes	No	Skip
5.7	From where/whom have you heard about the names of these methods?		Yes	No	
		Health worker visited home in here	1	0	
		Doctor/Nurse from Barma	1	0	
		Doctor/ Nurse in here	1	0	
		Family Member (e.g. sister/mother/sister-in-law etc.)	1	0	
		Neighbor	1	0	
		Women Friendly Center	1	0	
		Teacher	1	0	
		Heard from others	1	0	
		Others (Specify)…………………. 96			
		Didn't answer ……………………99			
5.8	When was the last time you used this (name of the method) method?	When I was in my country…………1<br>Currently Using ……………….… 2<br>1-2 months ago ………………..…3<br>3-6 months ago.………………..…4<br>more than 6 months ago.………...5<br>Not applicable.…………………98<br>Didn't answer ……………………99			Currently Using → Next section
5.9	Why did you stop using this (name of the method) method?<br><br>Multiple Response		Yes	No	
		Don't know where to get	1	0	
		Had physical problem	1	0	
		Husband didn't allow any more	1	0	
		Wanted to conceive child	1	0	
		Others (Specify)………………………96			

Section 6: Menstrual Regulation (MR) and abortion

No.	Questions and instructions	Coding categories	Yes	No	Skip
**6.1**	Have you ever had a pregnancy that ended using menstrual regulation or was aborted after you came here?	Yes ………………………………1<br>No …………………………………2<br>Didn't answer ……………………99			No → Next section
**6.2**	How did you do the Menstrual Regulation?<br><br>Multiple Question		Yes	No	
		Through Medicine	1	0	

ID of the Participant: |___|___|___|___|___|___|___|___|

For peer review only - http://bmjopen.bmj.com/site/about/guidelines.xhtml

Survey Questionnaire
Situation Analysis for Delivering Integrated Comprehensive SRHR Services for Rohingya Refugees in Cox's Bazar, Bangladesh

		Went to Doctor	1	0	
		Went to traditional healer	1	0	
		Through TBA	1	0	
		Through machine	1	0	
		Others (Specify)…………………. 96			
**6.3**	How many times did you successfully terminate a pregnancy?	Number of times |__|__|			
**6.4**	When was the last time you terminated pregnancy? [*If they answer in months then multiply the number of months by 30*]	months |__|__|			
**6.5**	Did you seek any help for such pregnancy termination in the last time?	Yes ………………………………1<br>No …………………………………2<br>Didn't answer ……………………99			No / Didn't answer → 6.7
**6.6**	From where you received help/services? (Instruction: If the respondent says any name, probe if that is healthcare facility)	Home ………………………………1<br>Healthcare Facility (Specify) ……..2<br>Others (Specify)…………………. 96<br>Didn't answer ……………………99			→ 6.8
**6.7**	(If you didn't seek service), What was the reason for not seeking help / service?<br><br>Multiple Response		Yes	No	
		Did not feel the necessity	1	0	
		Did not know where/who to go	1	0	
		Did not know such care	1	0	
		Health center is far/Access to facility was/is difficult	1	0	
		Services were/are not available here	1	0	
		Did not like services provided in the facilities	1	0	
		Family/Husband did not allow	1	0	
		Did not have money	1	0	
		Nobody to accompany	1	0	
		Previous experience is not good	1	0	
		Concerned about being treated by a male doctor	1	0	
		Afraid to go health care center	1	0	
		Hadn't time	1	0	
		Neighbor forbade to go	1	0	
		Health care center was far away from home	1	0	
		Others (Specify) ……………………96			
		Didn't Answer ………………………99			
**6.8**	Was there any sort of complication related to your last pregnancy termination?	Yes ………………………………1<br>No …………………………………2<br>Didn't answer ……………………99			No / Didn't answer → Next section
**6.9**	What were the complications?<br><br>Multiple Response		Yes	No	
		Hemorrhage/vaginal bleeding	1	0	
		Incomplete MR	1	0	
		Infection	1	0	

ID of the Participant: \|___\|___\|___\|___\|___\|___\|___\|___\|

For peer review only - http://bmjopen.bmj.com/site/about/guidelines.xhtml

Survey Questionnaire
Situation Analysis for Delivering Integrated Comprehensive SRHR Services for Rohingya Refugees in Cox's Bazar, Bangladesh

		Headache	1	0	
		Vomiting	1	0	
		Fever	1	0	
		Abdominal pain	1	0	
		Irregular menstruation	1	0	
		Weakness	1	0	
		Others (Specify)………………… 96			
		Didn't answer ……………………99			
**6.10**	Did seek any help for the complication (s)?	Yes ………………………………1<br>No …………………………………2<br>Didn't answer ……………………99			No, Didn't answer → 6.12
**6.11**	From where you received help/services?<br>(Instruction: If the respondent says any name, probe if that is healthcare facility)	Home ………………………………1<br>Healthcare Facility (Specify) ……...2<br>Others (Specify)………………… 96<br>Didn't answer ……………………99			Next section
**6.12**	(If you didn't seek service), What was the reason for not seeking help / service?<br><br>Multiple Response		Yes	No	
		Did not feel the necessity	1	0	
		Did not know where/who to go	1	0	
		Did not know such care	1	0	
		Health center is far/Access to facility was/is difficult	1	0	
		Services were/are not available here	1	0	
		Did not like services provided in the facilities	1	0	
		Family/Husband did not allow	1	0	
		Did not have money	1	0	
		Nobody to accompany	1	0	
		Previous experience is not good	1	0	
		Concerned about being treated by a male doctor	1	0	
		Afraid to go health care center	1	0	
		Hadn't time	1	0	
		Neighbor forbade to go	1	0	
		Health care center was far away from home	1	0	
		Others (Specify) ……………………96			
		Didn't Answer ………………………99			

Section 7: Sexually Transmitted Diseases

No.	Questions and instructions	Coding categories			Skip
7.1	There are some diseases that transmitted through sexual act/intercourse. Have you heard about of any of such diseases?	Yes ………………………………1<br>No …………………………………2<br>Didn't answer ……………………99			
7.2	Have you heard the name of HIV/AIDS?	Yes ………………………………1<br>No …………………………………2			
7.3	Have you recently had any of these symptoms after coming here?		Yes	No	

ID of the Participant: |___|___|___|___|___|___|___|___|

For peer review only - http://bmjopen.bmj.com/site/about/guidelines.xhtml

Survey Questionnaire
Situation Analysis for Delivering Integrated Comprehensive SRHR Services for Rohingya Refugees in Cox's Bazar, Bangladesh

No.	Questions and instructions	Coding categories			Skip
	(*read out one by one and mark all she mentioned*)<br><br>MULTIPLE RESPONSE	Vaginal itching	1	0	
		Vaginal blisters or blisters in the genital area (the region covered by underwear)	1	0	
		Vaginal rash or rash in the genital area	1	0	
		Burning urination	1	0	
		Painful urination	1	0	
		Pain during intercourse	1	0	
		Bleeding or spotting between menstrual cycles	1	0	
		Painless ulcers on the vagina	1	0	
		Pelvic pain	1	0	
		Rectal pain, bleeding, or discharge (after receiving anal sex)	1	0	
		No symptoms	1	0	→ Next Section
		Never had physical relation	1	0	
		Others ……………………………96			
		Not Applicable ……………………98			
7.4	Did you seek any treatment for any of the symptom you just mentioned?	Yes ……………………………….1<br>No ………………………………….2 →<br>Didn't answer ……………………99 →			7.6<br>Next Section
7.5	Where did you seek treatment?<br>(Instruction: If the respondent says any name, probe if that is healthcare facility)	Home ……………………………….1<br>Healthcare Facility (Specify) ……...2<br>Others (Specify)…………………. 96<br>Didn't answer ……………………99			
7.6	(If you didn't seek treatment), What was the reason for not seeking treatment?		Yes	No	
		Did not feel the necessity	1	0	
		Did not know where/who to go	1	0	
		Did not know such care	1	0	
		Health center is far/Access to facility was/is difficult	1	0	
		Services were/are not available here	1	0	
		Did not like services provided in the facilities	1	0	

ID of the Participant: |___|___|___|___|___|___|___|___|

For peer review only - http://bmjopen.bmj.com/site/about/guidelines.xhtml

Survey Questionnaire
Situation Analysis for Delivering Integrated Comprehensive SRHR Services for Rohingya Refugees in Cox's Bazar, Bangladesh

No.	Questions and instructions	Coding categories			Skip
		Family/Husband did not allow	1	0	
		Did not have money	1	0	
		Nobody to accompany	1	0	
		Previous experience is not good	1	0	
		Concerned about being treated by a male doctor	1	0	
		Afraid to go health care center	1	0	
		Hadn't time	1	0	
		Neighbor forbade to go	1	0	
		Health care center was far away from home	1	0	
		Others (Specify)………………………. 96			
		Didn't answer ………………………..99			

Section 8: Service utilization and barriers

No.	Questions and instructions	Coding categories			Skip
8.1	Have you ever received any SRH service (inclusive any of the above or for other services) for yourself from any health worker or doctor or health center?	Yes ……………………………….1<br>No ………………………………….2<br>Didn't answer ……………………99			 → 8.11<br>→ End
8.2	What services had you received? MULTIPLE RESPONSE		Yes	No	
		Contraceptive method	1	0	
		Antenatal care	1	0	
		Delivery care	1	0	
		Postnatal care	1	0	
		MR/post abortion care	1	0	
		STI/STD	1	0	
		Others (Specify)………………………...96			
		Did not answer…………………………99			
8.3	From who/where had you received services? (Instruction: If the respondent says any name, probe if that is healthcare facility)	Home ……………………………….1<br>Healthcare Facility (Specify) ……...2<br>Others (Specify)………………… 96<br>Didn't answer ……………………99			→ End
8.4	Why did you choose that facility? MULTIPLE RESPONSE		Yes	No	
		Close to my house	1	0	
		Suggested by husband	1	0	
		Suggested by other family member	1	0	
		Suggested by Neighbors	1	0	
		Recommended by health workers visited home	1	0	
		Recommended by Majhi	1	0	
		Doctor/ Nurse suggested	1	0	
		NGO suggested	1	0	
		Not Applicable	1	0	
		Others (Specify)……………………….96			
		Did not answer…………………………99			

ID of the Participant: |___|___|___|___|___|___|___|___|

For peer review only - http://bmjopen.bmj.com/site/about/guidelines.xhtml

Survey Questionnaire
Situation Analysis for Delivering Integrated Comprehensive SRHR Services for Rohingya Refugees in Cox's Bazar, Bangladesh

No.	Questions and instructions	Coding categories			Skip
8.5	Now I want to know about your experience with the services you received.<br>How long did it take to go to the health facility?	Less than 15 minutes ……………….1<br>15 to 30 minutes………………….2<br>30 minutes to 1 hour…………..…3<br>1 hour to 2 hour…………………4<br>more than 2 hours………………..5<br>Not Applicable…………………..6			
8.6	How did you go there?		Yes	No	
		By walking	1	0	
		By rickshaw/van	1	0	
		Ambulance	1	0	
		CNG	1	0	
		Tomtom	1	0	
		Bus	1	0	
		Hemorrhage/vaginal bleeding	1	0	
		Not Applicable	1	0	
		Others (Specify)………………………96			
		Did not answer…………………………99			
8.7	How long did you had to wait at the facility for getting the services (after entering to facility to receive service)?<br>*If they respond in hours please convert it in minutes*	Minutes |__|__|			
8.8	Did you feel comfortable enough to explain your problem and ask questions to the service provider?	Yes ………………………………1<br>No ……………………………….2<br>Didn't answer……………………… 99			→8.10 (Yes)
8.9	Why didn't you feel comfortable?<br><br>MULTIPLE RESPONSE		Yes	No	
		Afraid of service provider	1	0	
		Didn't understand language	1	0	
		Felt shy	1	0	
		There was no privacy	1	0	
		Service provider was male	1	0	
		Service provider behaved badly	1	0	
		Service provider didn't ask	1	0	
		The center was very crowded	1	0	
		Environment was unfamiliar	1	0	

ID of the Participant: |___|___|___|___|___|___|___|___|

For peer review only - http://bmjopen.bmj.com/site/about/guidelines.xhtml

Survey Questionnaire
Situation Analysis for Delivering Integrated Comprehensive SRHR Services for Rohingya Refugees in Cox's Bazar, Bangladesh

No.	Questions and instructions	Coding categories			Skip
		There was no seating arrangement	1	0	
		Service provider was in rush and didn't allow time	1	0	
		Others (Specify)…………………. 96			
8.10	What did you like about the facility you visited?<br><br>MULTIPLE RESPONSE		Yes	No	
		Clean Place	1	0	
		Good behavior of service providers	1	0	
		Close to home	1	0	
		Provided medicine	1	0	
		Easy to access	1	0	
		Providers understand my language	1	0	
		Female provider examined	1	0	→ End
		Less waiting time	1	0	
		Maintain privacy	1	0	
		Good treatment	1	0	
		Others (Specify) ……..…………….96			
		Not Applicable ……………….....….97			
		Did Not Answer ………………..…....99			
8.11	What are the reasons that you did not receive services from any health facility/ health worker?<br><br>MULTIPLE RESPONSE		Yes	No	
		Did not feel the necessity	1	0	
		Did not know where/who to go	1	0	
		Did not know such care	1	0	
		Health center is far/Access to facility was/is difficult	1	0	
		Services were/are not available here	1	0	
		Did not like services provided in the facilities	1	0	
		Family/Husband did not allow	1	0	
		Did not have money	1	0	
		Nobody to accompany	1	0	
		Previous experience is not good	1	0	

ID of the Participant: |___|___|___|___|___|___|___|___|

For peer review only - http://bmjopen.bmj.com/site/about/guidelines.xhtml

Survey Questionnaire
Situation Analysis for Delivering Integrated Comprehensive SRHR Services for Rohingya Refugees in Cox's Bazar, Bangladesh

No.	Questions and instructions	Coding categories			Skip
		Concerned about being treated by a male doctor	1	0	
		Afraid to go health care center	1	0	
		Hadn't time	1	0	
		Neighbor forbade to go	1	0	
		Health care center was far away from home	1	0	
		Others (Specify)………………………..96			
		Did not answer…………………………99			

For peer review only

ID of the Participant: |___|___|___|___|___|___|___|___|

For peer review only - http://bmjopen.bmj.com/site/about/guidelines.xhtml

Situation Analysis for Delivering Integrated Comprehensive SRHR Services for Rohingya Refugees in Cox's Bazar, Bangladesh

Facility Assessment

Conducted by
BRAC James P Grant School of Public Health (JPGSPH), BRAC University
Funded by
World Health Organization (WHO)

Questionnaire and Checklists
(For Health Facilities)

A1. Unique identification number of the facility	
A2. Serial number of the facility	
A3. Name of the Upazila	
A4. Name of the camp/area	
A5. Name of the health facility	
A6. Date of the assessment	
A7. Starting time of the assessment	
A8. Ending time of the assessment	
A9. Name of the Respondent, Designation & Cell number	
A10. Name of the assessor	
A11. Signature of the assessor	

For peer review only - http://bmjopen.bmj.com/site/about/guidelines.xhtml

1. General Information

1.1 Types of the facility (Please circle the appropriate one)

a. Community clinic
b. Health post
c. Primary Health Center (PHC)
d. National non-government organization (NNGO) Hospital
e. International non-government organization (INGO)Hospital
f. Other (Specify)

1.2 Management of the facility (Please circle the appropriate one)

a. Government organization (GO) (Specify) ______________________________
b. National non-government organization (NNGO) (Specify) ___________________________
c. International non-government organization (INGO) (Specify) _______________________
d. Ownership by more than one organization (Specify) (Specify) _______________________
e. Other (Specify) ________________________

1.3 Source of external Financial/resource support if applicable (Please circle the appropriate one)

a. Government organization (GO) (Specify) ______________________________
b. National non-government organization (NNGO) (Specify) ___________________________
c. International non-government organization (INGO) (Specify) _________________________
d. Multi-donor (Specify) __________________________
e. Other (Specify) __________________________

1.4 How many days per week is the facility opened? (Please circle the appropriate one)

a. 7 days a week
b. 6 days a week
c. 5 days a week
d. Others (Specify)

1.5 What is your Working hours? (Please circle the appropriate one)

a. 24 hours
b. 9 am - 4 pm
Other (specify

1.6 Estimated number of population served by your facility: __________

For peer review only - http://bmjopen.bmj.com/site/about/guidelines.xhtml

2. Health service information

2.1 Availability of sexual and reproductive health services

Serial no.	Name of the services	Code<br><br>1 = Yes<br>0 = Not available	Designation of the service provider/s<br>Code: 1, 2, 3, 4, 5, 6, 7, 8, 9, 97<br>*(for the details of this code follow the attached codebook)*	If the service is not available, then do you refer the patients?<br>Code<br>1 = Yes<br>0 = No	If yes, write the referred place/s<br>*(Most frequently referred)*
1.	ANC				
1.1	**Obstetric and foetal assessment** –<br>• Maternal weight<br>• BP measurement<br>• Oedema<br>• Fundal height<br>• Foetal heartbeat				
1.2	**Screening and tests**<br>• Urinalysis<br>• Hb estimation<br>• Blood grouping and Rh typing<br>• Testing for HIV, syphilis<br>• Blood sugar<br>• Ultrasonogram (referred cases for suspicion of low foetal growth				
1.3	**Identify & manage obstetric emergencies**<br>• Pre/eclampsia<br>• Ante-partum Haemorrhage<br>• Abdominal pain<br>• Premature rupture of membranes				
1.4	**Health education, advice, and counselling** (Nutrition,				

For peer review only - http://bmjopen.bmj.com/site/about/guidelines.xhtml

Serial no.	Name of the services	Code<br><br>1 = Yes<br>0 = Not available	Designation of the service provider/s<br>Code: 1, 2, 3, 4, 5, 6, 7, 8, 9, 97<br>*(for the details of this code follow the attached codebook)*	If the service is not available, then do you refer the patients?<br>Code<br>1 = Yes<br>0 = No	If yes, write the referred place/s<br>*(Most frequently referred)*
	Complications of pregnancy / danger sign, Rest, Birth plan and emergency plan)				
2.	Eclampsia Management				
3.	PPH (Postpartum Hemorrhage) Management				
4.	Maternal Immunization				
5.	Delivery Care<br>• Normal Vaginal Delivery<br>• C-section<br>• Deliveries that require vacuum extraction assistance				
6.	Initial stabilization of obstetric emergency before referral				
7.	Blood transfusion				
8.	Essential new born care				
9.	Post Natal Care				
9.1	Counselling on postnatal care, breastfeeding, etc.				

For peer review only - http://bmjopen.bmj.com/site/about/guidelines.xhtml

Facility mapping tool
SRHR WHO Project

Serial no.	Name of the services	Code<br><br>1 = Yes<br><br>0 = Not available	Designation of the service provider/s<br><br>Code: 1, 2, 3, 4, 5, 6, 7, 8, 9, 97<br>*(for the details of this code follow the attached codebook)*	If the service is not available, then do you refer the patients?<br><br>Code<br><br>1 = Yes<br>0 = No	If yes, write the referred place/s<br><br>*(Most frequently referred)*
9.2	Post-Natal clinical history (pain, fever, haemorrhage)				
9.3	Identification and management of post-natal complications:<br>-Anaemia<br>-Puerperal psychosis				
9.4	Identification and management of obstetric complications:<br>-Haemorrhage<br>-Puerperal infection/sepsis				
9.5	Supply of Iron and Folic Acid				
9.6	Counselling and Provision of FP methods				
10.	Family Planning				
11.	FP services: Pill				
12.	FP services: Male Condoms				
13.	Emergency Contraception				
14.	FP services: Injection				
15.	FP services: Implant/Norplant				
16.	FP services: IUD				

For peer review only

Serial no.	Name of the services	Code<br><br>1 = Yes<br>0 = Not available	Designation of the service provider/s<br>Code: 1, 2, 3, 4, 5, 6, 7, 8, 9, 97<br>*(for the details of this code follow the attached codebook)*	If the service is not available, then do you refer the patients?<br>Code<br>1 = Yes<br>0 = No	If yes, write the referred place/s<br>*(Most frequently referred)*
17.	FP services: Vasectomy				
18.	FP services: Tubectomy				
19.	STD/RTI Management				
20.	HIV AIDS Counseling				
21.	HIV AIDS Testing				
22.	Prevention of maternal –to-child transmission of HIV (PMTCT) *(one kind of medicine to stop transmitting HIV to the newborn)*				
23.	Menstrual regulation (MR)				
24.	Management of miscarriage and complications of abortions				
25.	Clinical Management of Rape (CMR)				
26.	Management of Psychosocial issues				
27.	Adolescent friendly health services (counseling on Sex education/ sexual health care)				
28.	Adolescent friendly health services				

For peer review only

Serial no.	Name of the services	Code<br>1 = Yes<br>0 = Not available	Designation of the service provider/s<br>Code: 1, 2, 3, 4, 5, 6, 7, 8, 9, 97<br>*(for the details of this code follow the attached codebook)*	If the service is not available, then do you refer the patients?<br>Code<br>1 = Yes<br>0 = No	If yes, write the referred place/s<br>*(Most frequently referred)*
	(counseling on FP services)				
29.	Adolescent friendly health services (counseling on HIV services)				
30.	Others				

2.2 If you don't provide the SRHR services, please mention the reason for not providing the SRHR services.

____
____
____

2.3 Human Resources: Key Staff information

Serial no.	Categories	No of staff	Job Nature<br>Code<br>1= Fulltime<br>2= Part-time<br>3= On-call
1.	Gynecologists / Gynae Consultant		
2.	Anesthesiologist		
3.	Residential Medical Officer (RMO)		

For peer review only - http://bmjopen.bmj.com/site/about/guidelines.xhtml

Serial no.	Categories	No of staff	Job Nature Code 1= Fulltime 2= Part-time 3= On-call
4.	Medical Officer		
5.	Medical Assistant		
6.	SACMO		
7.	Health Inspector		
8.	Nurse		
9.	Midwife		
10.	Trained Birth Attendants (TBAs)		
11.	Family Welfare Assistant (FWA)		
12.	Community Health Care Provider (CHCP)		
13.	Psycho-social Counsellor		
14.	Laboratory technicians/ Medical Technologist		
97.	Others (Specify)		

2.4 Training

If any of your staff received any of the following training:

Serial No.	Types of Training (RECEIVED IN THE PAST Three MONTHS)	Code 1 = Yes 0 = No	Who received it (Doctor, Nurse, Midwifes etc?) Code: 1 – 20, 97 *(for the details of this code follow the attached codebook)*
1.	Helping Babies Breathe		
2.	Emergency Response		
3.	Helping Mothers Survive		

For peer review only - http://bmjopen.bmj.com/site/about/guidelines.xhtml

4.	Gender Based Violence		
5.	Clinical Management of Rape (CMR)		
6.	OGSB Training - FP (Long Acting & Permanent)		
7.	EmONC		
8.	OGSB Training - Infection Prevention Control		
9.	OGSB Training – RTI & STI		
10.	OGSB Training – Management & Care of Maternal & Child Nutrition		
11.	ANC Package		
12.	MR & PAC		
13.	Orientation on community distribution of misoprostol		
14.	MISP Training for Coordinator		
15.	OGSB Training – Safe Delivery Practice		
16.	Labor Room Protocol		
17.	Cervical Cancer Screening		
18.	FP Counselling		
19.	IUD		
20.	Implant		
21.	PPIUD		
22.	Others		

2.4.1 Do you think is there any other training that you/your staff need to receive?

a. No = 0

b. Yes = 1 If Yes, specify ____

For peer review only - http://bmjopen.bmj.com/site/about/guidelines.xhtml

2.5 Information on total number of services

2.5.1 On average total number of Outpatient served in a day?

2.5.2 Total number of inpatient served in a month? (Last month/3 month average)

2.5.3 Information on total number of services

Serial no.	Services and Quantity	July 2018	August 2018	September 2018
1.	No. of total patients served			
2.	No. of ANC			
3.	Eclampsia Management			
4.	PPH (Postpartum Hemorrhage) Management			
5.	Total delivery			
	Normal Delivery			
	C section			
6.	No. of PNC			
7.	Blood Transfusion			
8.	No. of FP counseling			
9.	FP services: Pill			
10.	FP services: Condom			
11.	FP services: Injection			
12.	FP services: Implant/Norplant			
13.	FP services: IUD			
14.	FP services: Vasectomy			
15.	FP services: Tubectomy			
16.	No. of STD/RTI screening			
17.	No. of STD/RTI treatment			
18.	No of HIV/AIDS counselling			
19.	No of HIV/AIDS screening			

For peer review only - http://bmjopen.bmj.com/site/about/guidelines.xhtml

Serial no.	Services and Quantity	July 2018	August 2018	September 2018
20.	No. of MR service			
21.	No. of management of miscarriage and complications of abortions			
22.	No of Post rape treatment			
23.	No. of adolescent health service			
24.	Total no. of referred patients			
25.	Other (Specify			

For peer review only

2.6 Availability of ambulance service

2.6.1 Is there any ambulance services in your facility?

a. Yes = 1
b. No = 0

2.6.2 If yes, then how many do you have? [ ]

2.6.3 If No, Then do you have access to other's (private/NGO/UN/govt.) ambulance services that you could use?

a. Yes = 1, Specify whose______________________________
b. No = 0

2.6.4 When do you refer patients, how do you transport them to the referred place? (Mode of transportation)

____ (Free Listing)

For peer review only - http://bmjopen.bmj.com/site/about/guidelines.xhtml

2.7 Structure of the facility /Availability of rooms (related to SRH)

Serial no.	Name of department/room	Code<br>1 = Yes<br>0 = Not available	Remark
1.	Emergency room		
2.	Consultant's room		
3.	Counselling room		
4.	Patient's waiting place		
5.	ANC/PNC room		
6.	Labor Room		
7.	Labor Ward		
8.	Women Ward		
9.	VIA/PAC room		
10.	Room for USG		
11.	Laboratory for lab tests		
12.	Room for blood transfusion		
13.	Maternity OPD		
14.	Breast feeding corner in OPD		
15.	General Operation Theatre		
16.	Dispensary		
17.	Other (Specify)		

2.8 Number of maternity beds at the facility

2.9 Number of beds occupied by the patients on average

For peer review only - http://bmjopen.bmj.com/site/about/guidelines.xhtml

3. Equipment, drugs and supplies

Serial No	KIT Number & Name	Received? Yes = 1 No = 0	If yes, How many? (Boxes)	If No, Then Why?	If not a UNFPA KIT receiver then how do you procure/ source those drugs and supplies
1	Kit 1: Condoms				
2	Kit 2: Clean Delivery, Individual				
3	Kit 3: Post Rape Treatment				
4	Kit 4 Oral And Injectable Contraception				
5	Kit 5: Treatment Of Sexually Transmitted Infections				
6	Kit 6: Clinical Delivery Assistance				
7	Kit 7: Intrauterine Device				
8	Kit 8: Management Of Miscarriage And Complications Of Abortion				
9	Kit 9: Suture Of Tears (Cervical & Vaginal Examination)				
10	Kit 10: Vacuum Extraction Delivery				
11	Kit 11: Referral Level Kit For Reproductive Health				
12	Kit 12 Blood Transfusion				

For peer review only - http://bmjopen.bmj.com/site/about/guidelines.xhtml

Infrastructure

a. Standard precautions for infection prevention (ref: SARA reference manual of WHO)

Serial no.	Activities	Code<br>1 = Yes<br>0 = Not available	Remarks *(How?)*
1.	Appropriate storage of sharp object wastes (sharps box/container)		
2.	Appropriate storage of infectious waste (waste receptacle with lid and plastic bin liner)		
3.	Safe final disposal of sharp objects		
4.	Safe final disposal of infectious wastes		

ANNEXURE

Code Book:

2.1 Health Service Information on SRHR Designation of the service provider/s Code: 1 – 20, 97 *(for the details please follow below mentioned list)*

1 = Gynecologists / Gynae Consultant

2 = Anesthesiologist

3 = Residential Medical Officer (RMO)

4 = Medical Officer

5 = Medical Assistant

6 = SACMO

7 = Health Inspector

8 = Nurse

9 = Midwife

10 = Trained Birth Attendants (TBA)

11 = other community health workers (FWA)

12 = CHCP

13 = Counsellor

14 = Laboratory technicians/ Medical Technologist

97 = Other (specify)

For peer review only

For peer review only - http://bmjopen.bmj.com/site/about/guidelines.xhtml

STROBE 2007 (v4) Statement—Checklist of items that should be included in reports of *cross-sectional studies*

Section/Topic	Item #	Recommendation	Reported on page #
**Title and abstract**	1	(*a*) Indicate the study's design with a commonly used term in the title or the abstract	1, 2
		(*b*) Provide in the abstract an informative and balanced summary of what was done and what was found	2
**Introduction**			
Background/rationale	2	Explain the scientific background and rationale for the investigation being reported	4, 5
Objectives	3	State specific objectives, including any prespecified hypotheses	5
**Methods**			
Study design	4	Present key elements of study design early in the paper	6
Setting	5	Describe the setting, locations, and relevant dates, including periods of recruitment, exposure, follow-up, and data collection	6, 7, 8, 9, 10, 11
Participants	6	(*a*) Give the eligibility criteria, and the sources and methods of selection of participants	6, 7, 8, 9, 10, 11
Variables	7	Clearly define all outcomes, exposures, predictors, potential confounders, and effect modifiers. Give diagnostic criteria, if applicable	6, 7, 8, 9, 10, 11
Data sources/ measurement	8*	For each variable of interest, give sources of data and details of methods of assessment (measurement). Describe comparability of assessment methods if there is more than one group	N/A
Bias	9	Describe any efforts to address potential sources of bias	10
Study size	10	Explain how the study size was arrived at	7, 8, 9, 11
Quantitative variables	11	Explain how quantitative variables were handled in the analyses. If applicable, describe which groupings were chosen and why	8, 11
Statistical methods	12	(*a*) Describe all statistical methods, including those used to control for confounding	8, 11
		(*b*) Describe any methods used to examine subgroups and interactions	8, 10, 11, 12
		(*c*) Explain how missing data were addressed	N/A
		(*d*) If applicable, describe analytical methods taking account of sampling strategy	7, 9, 10, 11
		(*e*) Describe any sensitivity analyses	N/A
**Results**			

For peer review only

Participants	13*	(a) Report numbers of individuals at each stage of study—eg numbers potentially eligible, examined for eligibility, confirmed eligible, included in the study, completing follow-up, and analysed	N/A
		(b) Give reasons for non-participation at each stage	N/A
		(c) Consider use of a flow diagram	N/A
Descriptive data	14*	(a) Give characteristics of study participants (eg demographic, clinical, social) and information on exposures and potential confounders	N/A
		(b) Indicate number of participants with missing data for each variable of interest	N/A
Outcome data	15*	Report numbers of outcome events or summary measures	N/A
Main results	16	(*a*) Give unadjusted estimates and, if applicable, confounder-adjusted estimates and their precision (eg, 95% confidence interval). Make clear which confounders were adjusted for and why they were included	N/A
		(*b*) Report category boundaries when continuous variables were categorized	N/A
		(*c*) If relevant, consider translating estimates of relative risk into absolute risk for a meaningful time period	N/A
Other analyses	17	Report other analyses done—eg analyses of subgroups and interactions, and sensitivity analyses	N/A
**Discussion**			
Key results	18	Summarise key results with reference to study objectives	N/A
Limitations	19	Discuss limitations of the study, taking into account sources of potential bias or imprecision. Discuss both direction and magnitude of any potential bias	2, 3, 13
Interpretation	20	Give a cautious overall interpretation of results considering objectives, limitations, multiplicity of analyses, results from similar studies, and other relevant evidence	N/A
Generalisability	21	Discuss the generalisability (external validity) of the study results	N/A
**Other information**			N/A
Funding	22	Give the source of funding and the role of the funders for the present study and, if applicable, for the original study on which the present article is based	14

*Give information separately for cases and controls in case-control studies and, if applicable, for exposed and unexposed groups in cohort and cross-sectional studies.

Note: An Explanation and Elaboration article discusses each checklist item and gives methodological background and published examples of transparent reporting. The STROBE checklist is best used in conjunction with this article (freely available on the Web sites of PLoS Medicine at http://www.plosmedicine.org/, Annals of Internal Medicine at http://www.annals.org/, and Epidemiology at http://www.epidem.com/). Information on the STROBE Initiative is available at www.strobe-statement.org.

For peer review only
